# Essential mitochondrial activity in *Physcomitrium patens* relies on complementary cytochrome and alternative oxidase pathways

**DOI:** 10.1111/tpj.71127

**Published:** 2026-09-17

**Authors:** Shun‐Ling Tan, Antoni M. Vera‐Vives, Heyu Wang, Xing Huang, Claudia Beraldo, Alessandro Alboresi, Tomas Morosinotto

**Affiliations:** ^1^ Department of Biology University of Padova Padova Italy; ^2^ Kunming Institute of Botany Chinese Academy of Sciences Kunming 650201 China

**Keywords:** AOX, cytochrome pathway, photosynthesis, ATP, *Physcomitrium patens*

## Abstract

Mitochondrial respiration catalyses the transfer of electrons from NADH to oxygen through the activity of five multiprotein complexes. In plants, mitochondria also possess additional alternative electron transport pathways, including the alternative oxidase (AOX) pathway, which transfers electrons from ubiquinol to O_2_, bypassing the cytochrome‐dependent electron transport chain. This study investigates the functional role of AOX during plant evolution by analysing *Physcomitrium patens* plants that lack or overexpress AOX. In the moss *P. patens,* AOX exhibits a remarkably high electron transport capacity, sufficient to fully compensate for the inactivation of the cytochrome pathway. Despite the high potential activity of AOX, *aox* knockout lines did not show significant defects in growth under various abiotic stresses. This suggests that the cytochrome pathway can compensate for AOX loss and effectively support the consumption of reducing power produced by photosynthesis even under stressful conditions. Although photosynthetically active cells can produce ATP in chloroplasts under illumination independently of mitochondrial electron transport, respiration is shown to be essential for the conversion of reducing power into ATP for distribution throughout the cell. Simultaneous inactivation of AOX and CIII was lethal, indicating that AOX and the complementary cytochrome pathway contribute to the essential role of mitochondrial respiration in the redox and energy balance in *P. patens* cells.

## Significance Statement

The AOX pathway in *Physcomitrium patens* has high electron transport capacity and shows high complementarity with the cytochrome pathway. The combined activities of AOX and cytochrome‐dependent respiration are essential for plant bioenergetics, contributing to ATP supply to the cell.

## INTRODUCTION

Plants rely on photosynthesis to convert sunlight into the chemical energy that supports their metabolism. The process involves a light‐dependent chloroplast electron transport chain (cETC), located in the thylakoid membrane to oxidise water, generating NADPH and ATP. These energy carriers are then exploited by the carbon assimilation phase, specifically the Calvin–Benson cycle in the chloroplast stroma, fixing CO_2_ into triose phosphates (Stitt et al., 2010). In natural environments, the rate of carbon fixation and associated metabolic pathways is continuously changing (Foyer et al., 2012; Ma et al., 2014; Walker et al., 2016), leading to highly dynamic demands for reducing power and ATP, in terms of quantity and balance.

In this context, photosynthetic organisms have evolved multiple mechanisms to modulate cETC and energy production to prevent imbalances that could lead to over‐reduction and photodamage. One key mechanism is cyclic electron transport around PSI (CEF), which redirects electrons from PSI back to the cytochrome *b6f* complex, producing additional ATP without synthesis of NADPH and helps prevent PSI over‐reduction under light stress (Tan et al., 2020; Yamori & Shikanai, 2016). Another route is pseudocyclic electron flow via flavodiiron proteins (FLV) or the Mehler reaction, where electrons are transferred to O_2_ producing H_2_O, supporting the generation of a proton motive force (Shimakawa et al., 2017) while acting as a safety valve to dissipate excess electrons in cETC (Gerotto et al., 2016). Additionally, non‐photochemical quenching (NPQ) is another important protective mechanism, which dissipates excess absorbed energy as heat (Demmig‐Adams et al., 2020).

Even if all energy ultimately originates from photosynthesis, respiration is an essential metabolic process in plants. Chloroplast and mitochondrial activities are tightly coordinated, and their interplay is essential for plant survival (Noctor et al., 2007; Raghavendra & Padmasree, 2003). Key physiological processes rely on organellar communication, such as photorespiration (Eisenhut et al., 2019), carbon and nitrogen metabolism (Medeiros et al., 2021; Smith et al., 2019; Vera‐Vives et al., 2025) and fatty acid synthesis (Guan et al., 2020). Furthermore, mitochondrial respiration was also shown to be essential for the supply of ATP to the cytosol of plant cells (Gardeström & Igamberdiev, 2016; Vera‐Vives et al., 2024; Voon et al., 2018).

The mitochondrial electron transport chain (mETC) in plants consists of five complexes (Complex I‐V) that mediate the transport of electrons from NADH and succinate to O_2_. CI and CII reduce ubiquinone (UQ) to ubiquinol (UQH_2_). The cytochrome (cyt) pathway, which consists of CIII (cyt *c* reductase) and CIV (cyt *c* oxidase), transports electrons from the UQ pool to O_2_. In CI, CIII, and CIV, electron transport is coupled with proton translocation across the inner mitochondrial membrane, generating a proton motive force (*pmf*) that is exploited by mitochondrial ATP synthase (CV) to synthesise ATP. In addition to these five complexes, plant mitochondria possess another pathway, mediated by alternative oxidase (AOX), which transfers electrons from ubiquinol to O_2_ bypassing two of the proton pumping sites, CIII and CIV (Millar et al., 2011; Vanlerberghe & McIntosh, 1997). Since AOX does not contribute to proton translocation, this pathway partially uncouples electron transport from ATP biosynthesis, thus decreasing respiratory energy yield per reducing equivalent. AOX activity was shown to support mitochondrial electron transport when the cytochrome pathway is inhibited or saturated, thereby preventing UQH_2_ pool over‐reduction and consequent reactive oxygen species (ROS) production, and thus constituting an electron security valve especially under stress conditions (Selinski et al., 2018). AOX activity by consuming excess reducing power was also shown to mitigate the risk of over‐reduction of photosynthetic electron transport (Florez‐Sarasa et al., 2020; Vanlerberghe et al., 2020; Vishwakarma et al., 2014). Due to its impact on ROS production and redox balance, AOX activity has also been associated with redox signalling networks (Van Aken, 2021; Van Aken et al., 2009; Vanlerberghe et al., 2020) and was shown to be induced by multiple stress conditions in plants (Arnholdt‐Schmitt et al., 2006; Clifton et al., 2006).

AOX is a highly conserved protein in the plant kingdom and is also present in fungi, protists, green algae and some animal species (Moore et al., 2013; Pennisi et al., 2016). In *Arabidopsis thaliana*, AOX is encoded by five nuclear genes (*AtAOX1a‐1d* and *AtAOX2*), with *AtAOX1a* being the most abundant isoform in all tissues (Clifton et al., 2006). *AtAOX1a* knockout plants were more sensitive than WT to low temperature (Fiorani et al., 2005), while its overexpression enhanced tolerance to salt stress (Smith et al., 2009). Short‐term high light treatment revealed that, when AOX was knocked out or inhibited by salicylhydroxamic acid (SHAM), Arabidopsis, tobacco and other C3 plants displayed increased UQ reduction levels and severe PSII photoinhibition (Yoshida, Watanabe, Hachiya, et al., 2011; Yoshida, Watanabe, Terashima, et al., 2011; Zhang et al., 2017). Various C4 plants, exposed to similar treatments, showed no detectable effect on PSII photoinhibition, suggesting species‐specific differences in AOX‐dependent photoprotection (Zhang et al., 2017). Under long term treatments, the growth of *AtAOX1a* knockout plants was not impaired compared with WT under both control and high light (Yoshida, Watanabe, Hachiya, et al., 2011), while the antisense suppression of *GmAOX2b* in soybeans led to impaired growth and reproductive development linked to reduced CO_2_ assimilation rate even under ambient condition (Chai et al., 2010, 2012). The role of AOX in modulating photosynthetic metabolism has also been demonstrated in unicellular photosynthetic organisms. In the diatom *Phaeodactylum tricornutum*, PtAOX contributed to optimising ATP/NADPH ratio in the chloroplasts and enhanced carbon fixation and growth (Bailleul et al., 2015). In *Chlamydomonas reinhardtii*, CrAOX1 was found to be critical for photosynthetic performance and survival at high irradiance by limiting ROS accumulation and further preventing PSII damage (Kaye et al., 2019; Mathy et al., 2010).

Although there is considerable evidence supporting the AOX role in modulating bioenergetic metabolism, its physiological impact appears to be highly differentiated in various species and growing conditions. In this context, it is interesting to investigate the role of AOX in the moss *Physcomitrium patens*, a nonvascular plant that diverged from the vascular plant ancestors early after land colonisation. Due to its evolutionary position and the specific features that connect aquatic and terrestrial lifestyles (Strotbek et al., 2013), the investigation of *P. patens* enables us to assess evolutionary differences and conserved trends during evolution and land colonisation. As another reason of interest, in the moss *Physcomitrium patens*, AOX is encoded by a single nuclear gene, and the lack of gene redundancy enables us to highlight its biological role (Neimanis et al., 2013).

To this end, in this study, we generated *PpAOX* knockout (*aox* KO) and overexpression lines (AOX OE) in *Physcomitrium patens* to explore the role of AOX and the interaction between mitochondria and chloroplast bioenergetic metabolism. Here, we show that AOX has a high electron transport capacity in *P. patens* and its overexpression reduces growth, possibly due to excessive energy waste. *aox* KO plants show limited differences compared to WT due to the efficient compensation by the cytochrome pathway. Inhibition of both pathways, however, is lethal showing that mitochondrial respiration, with AOX contribution, plays an essential role in photosynthetic cell metabolism, with a major impact on ATP supply to the cytosol.

## RESULTS

### AOX has high electron transport capacity in the moss *Physcomitrium patens*

The expected subcellular localisation of AOX in *P. patens* was first verified by transient expression of AOX‐YFP fusion protein in WT protoplasts. Microscopy analysis showed that AOX‐YFP did not colocalise with chlorophyll fluorescence and exhibited the distribution characteristic of a mitochondrial protein (Figure S1), confirming previous studies (Wu et al., 2019). To investigate the functional role of AOX, multiple independent *aox* KO plants and overexpressing plants (AOX OE) were generated by homologous recombination‐mediated gene targeting. Rescue lines were also generated by overexpressing AOX in *aox* KO background. RT‐PCR confirmed the expected expression of AOX in all the transformed lines (Figure S2).

**Figure 1:**
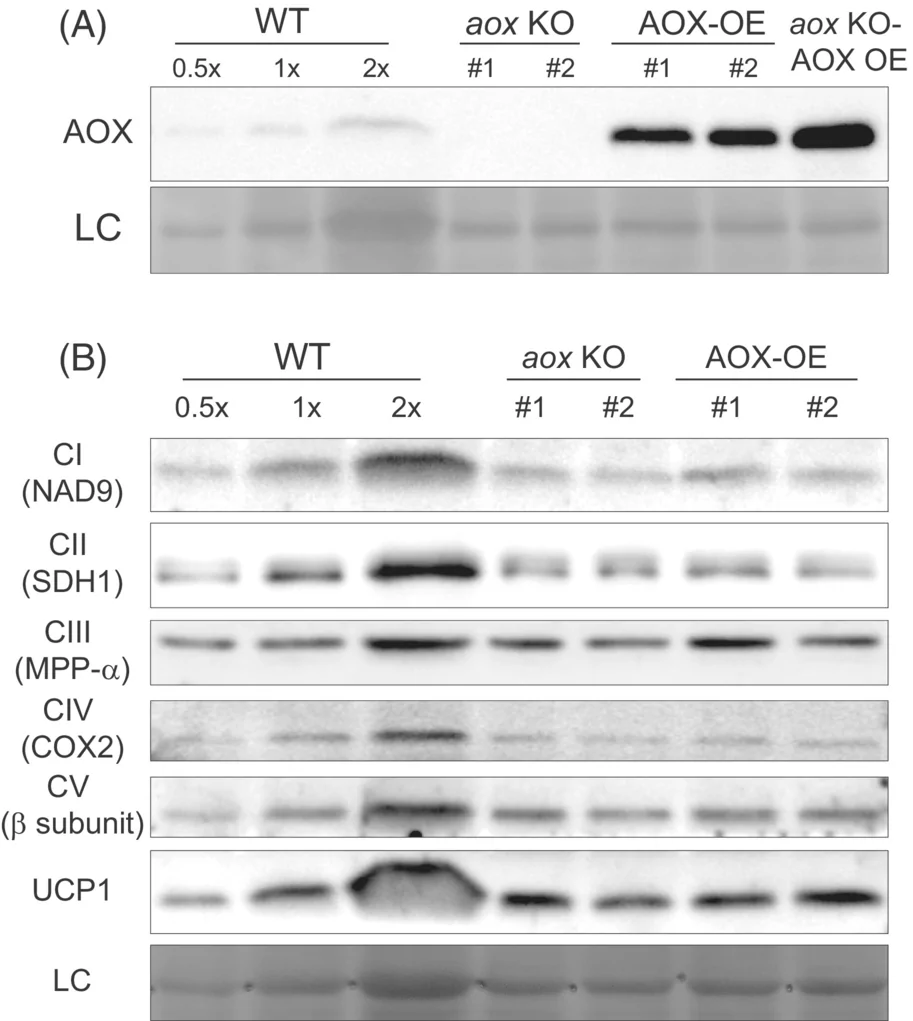
Immunoblot analysis of AOX and mitochondrial respiratory complex proteins in *Physcomitrium patens*. (A) AOX protein accumulation in WT, two independent *aox* KO lines, two independent AOX OE lines and one rescue line (*aox* KO complemented with AOX OE). Three dilutions of total protein extracts were loaded for WT (0.5, 1 and 2 μg of chlorophyll) while 1 μg of chlorophyll was loaded for all other lines. RuBisCO large subunit band is shown as loading control (LC). (B) Impact of alteration of AOX (*aox* KO and AOX OE) on the accumulation of other mitochondrial respiratory complexes. Total protein extracts equivalent to 0.5, 1 and 2 μg of chlorophyll were loaded for WT, while 1 μg of chlorophyll equivalent was loaded for all other lines. RuBisCO large subunit band is shown as representative loading control.

Protein accumulation in isolated lines was assessed using a specific anti‐AOX antibody (Figure 1A). In the KO lines, the accumulation of AOX was undetectable, while protein levels in AOX OE1 and AOX OE2 were more than ten times higher as compared to the WT plants, also confirming the recovery of protein accumulation in the rescue line (Figure 1A). Immunoblot analysis using specific antibodies against different subunits of mitochondrial respiratory complexes revealed no drastic changes in the accumulation of other respiratory complexes, except AOX (Figure 1B).

**Figure 2:**
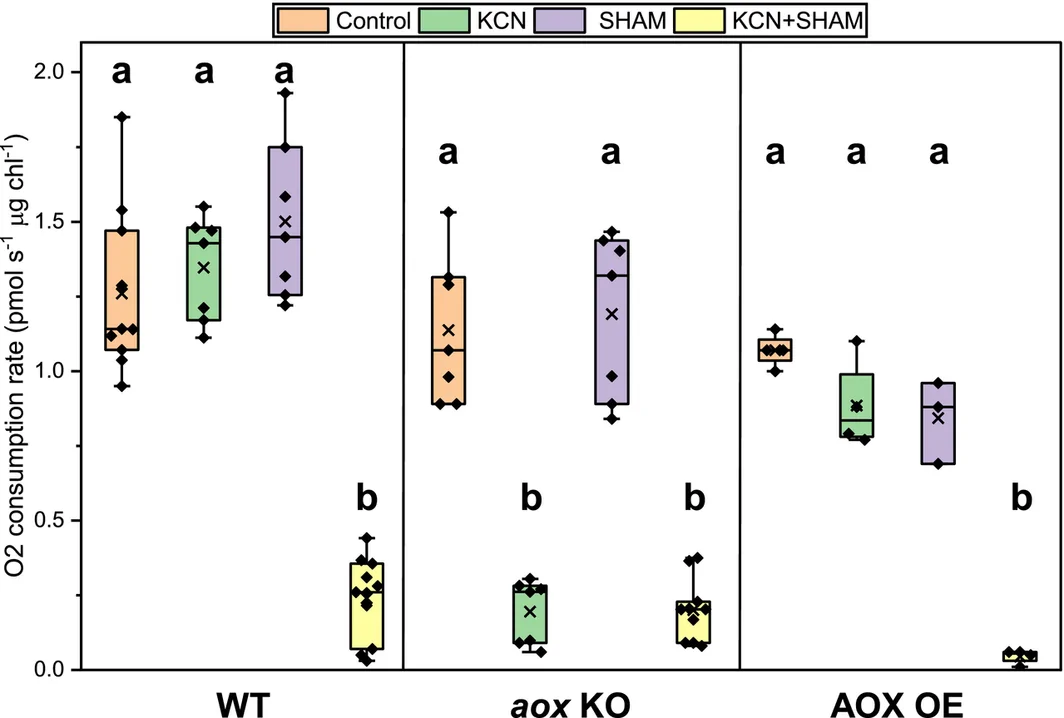
Effect of AOX on respiration in *Physcomitrium patens* protonema. After 40 min of dark adaptation, O_2_ consumption rate was measured in darkness for 10 min. Respiration was monitored under three conditions: in the absence of inhibitors (control; orange), in the presence of 1 mm KCN (green) and SHAM (purple), and both (yellow) to estimate capacity of cytochrome and alternative pathways (Mellon et al., 2021). Bars represent the average ± SD (*n* ≧ 4). *aox* KO reports pooled data of *aox*‐1 and *aox*‐2 independent lines. AOX OE reports pooled data of OE‐1 and OE‐2 independent lines. The different letters indicate statistically significant differences between different treatments within each genotype (WT and *aox* KO, one‐way anova, *P* < 0.05).

To quantify the impact of AOX on respiratory activity, the rate of O_2_ consumption in the dark was assessed, also using inhibitors of CIV (KCN) and AOX (SHAM) to estimate the capacity of cytochrome and alternative pathways. Both inhibitors showed no effect on the O_2_ consumption of WT plants, while respiration was almost completely inhibited when both were applied together (Figure 2) as previously observed (Vera‐Vives et al., 2025).

Similar measurements in *aox* KO plants did not show differences in respiratory activity compared to WT, and SHAM treatment had no effect on *aox* KO plants (*n* ≧ 4, *P* < 0.05) (Figure 2). However, in the presence of KCN alone, respiratory activity was inhibited to the same extent as in WT and *aox* KO plants treated with both KCN and SHAM together (Figure 2). AOX OE showed a similar trend to the WT with impacted respiration only when both KCN and SHAM were added. These results confirmed that the AOX pathway was inactive in *aox* KO but also suggested that both the cytochrome and alternative pathways had a high capacity in WT plants and that each of them was able, alone, to sustain full respiratory activity, even if the other was inhibited (Vera‐Vives et al., 2025). Under the conditions tested here, respiratory activity was likely limited by other factors and not by CIII or AOX, as also confirmed by the phenotype of AOX OE.

### Overexpression of AOX negatively impacts *Physcomitrium patens* growth

**Figure 3:**
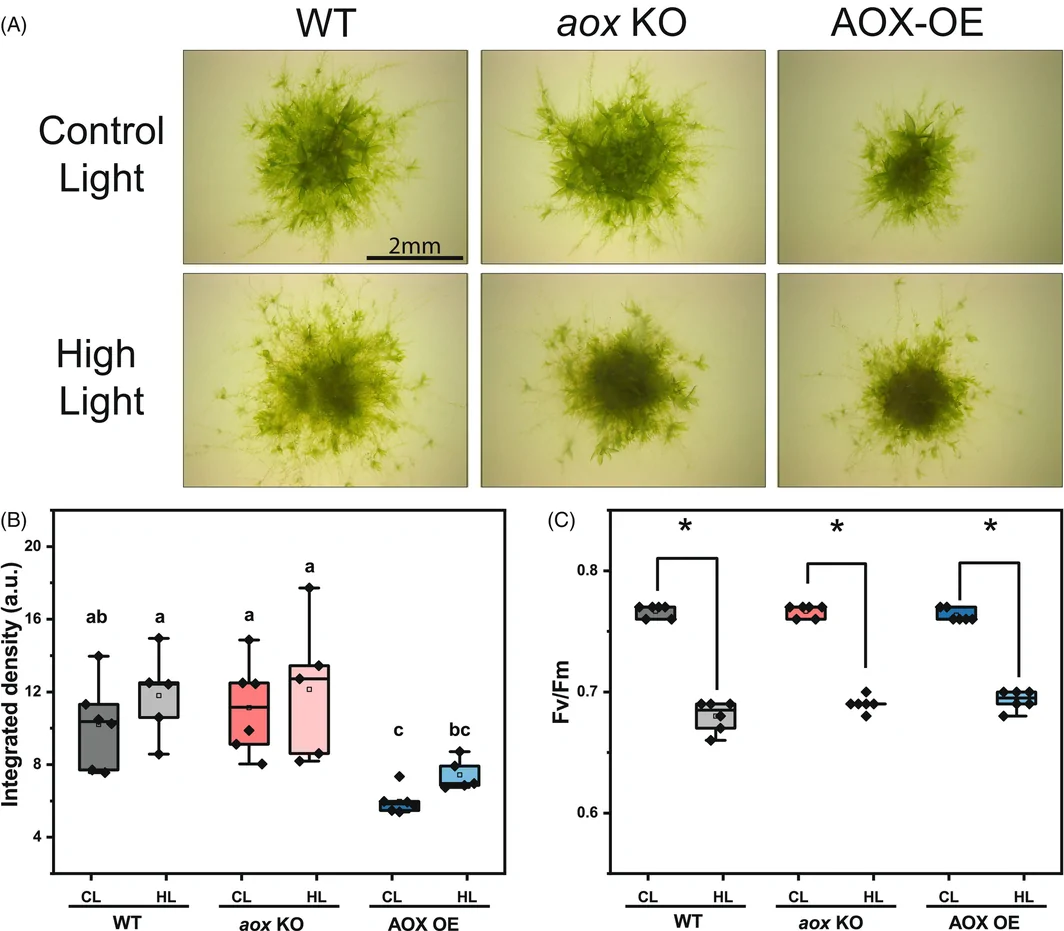
Growth phenotype of *P. patens* plants under control light and high light. 10‐day‐old protonema with a 2‐mm diameter were transferred on fresh PPNO_3_ medium and grown under control and high light (50 and 500 μmol photons m^−2^ sec^−1^, respectively) for 21 days. Representative images (A), growth quantification (B) and PSII maximum efficiency (C) of 21‐day‐old colonies. Data are shown as average ± SD (*n* ≧ 4). Bar = 2 mm. The different letters indicate statistically significant differences between different treatments in WT, *aox* KO plants and AOX OE plants, respectively (one‐way anova, *P* < 0.05). The asterisk indicates statistically significant differences between growth conditions in each genotype (independent sample test, *P* < 0.05). *aox* KO and AOX OE report pooled data of at least two independent lines.

The physiological impact of AOX was assessed by growing WT, *aox* KO, and AOX OE plants under control light conditions (50 μmol photons m^−2^ sec^−1^, Figure 3A). *aox* KO plants were indistinguishable from WT, while AOX OE exhibited a significant growth penalty (*n* ≧ 4, *P* < 0.05) (Figure 3A,B).

The plants were also exposed to various abiotic stresses to evaluate the potential impact of AOX in those conditions. Under strong illumination (500 μmol photons m^−2^ sec^−1^), growth of WT and *aox* KO plants remained comparable, with no significant differences (Figure 3A). AOX OE grew better under HL than CL but remained smaller than WT (Figure 3A,B). All three genotypes also showed a similar decrease in *Fv/Fm* under excess light treatment (*n* ≧ 4, *P* < 0.05) (Figure 3C). Based on previous results with *Arabidopsis thaliana*, wheat and rice (Bartoli et al., 2005; Challabathula et al., 2022; Giraud et al., 2008), we further examined the response to salt (100–400 mm NaCl) and cold (4°C) stress. The experimental conditions were selected to be severe enough to cause a reduction in *Fv/Fm* and a growth inhibition, but again no differences were observed between WT and *aox* KO plants (Figure S3). Although it is possible and even likely that different stresses or harsher/milder conditions could yield different results, the ones tested were sufficiently stressful to induce measurable growth reduction and photoinhibition and yet the absence of AOX had no measurable impact, while AOX OE plants did not show rescue or exacerbation of the phenotype (Figure S3).

### AOX absence has a limited impact on *Physcomitrium patens* photosynthetic properties, but its overexpression affects carbon fixation

To determine whether AOX accumulation affects photosynthetic electron transport, photosynthetic properties were analysed using PAM fluorometry under growth light (63 μmol photons m^−2^ sec^−1^) and saturating light intensity (330 μmol photons m^−2^ sec^−1^). Both *aox* KO mutants and AOX OE lines were indistinguishable from WT plants in all light intensities and considering all measured parameters, including PSI and PSII efficiency, the plastoquinone pool (PQ) and PSI redox state and activation of photoprotective mechanisms such as NPQ (Figures S4 and S5).

*In silico* analysis showed that *AOX* expression is higher in gametophores than in protonema (Figure S6), the tissue analysed in the above results. Immunoblotting confirmed that, while CI, CV and the uncoupling protein (UCP1) accumulated similarly in both developmental stages, CII was less abundant in gametophore and AOX and CIII showed higher accumulation (Figure S6). To assess whether high AOX and CII accumulation corresponded to a more prominent functional role, fluorescence measurements were repeated on WT and *aox* KO mutant gametophores. However, in similar fashion to protonema, no major differences were observed in *aox* KO plants but for a small reduction in NPQ activation and higher PSII efficiency under increasing light intensity (Figure S7).

**Figure 4:**
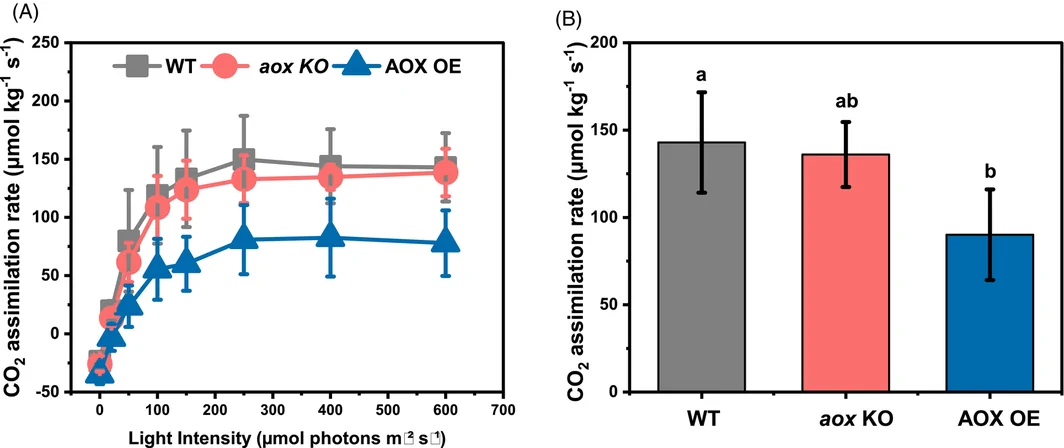
Carbon fixation capacity of *Physcomitrium patens*. (A) Light response curve of CO_2_ assimilation rate. (B) Steady‐state CO_2_ assimilation rate at saturated light intensity (300 μmol photons m^−2^ sec^−1^). Gametophores were grown under control light (50 μmol photons m^−2^ sec^−1^) for 30‐day‐old and then were measured with Li‐cor 6800. WT, *aox* KO and AOX OE lines are shown in black, red and blue, respectively. Average ± SD (*n* = 4) are reported. The different letters indicate statistically significant differences in WT, *aox* KO plants and AOX OE plants, respectively (one‐way anova, *P* < 0.05). *aox* KO and AOX OE report pooled data of at least two independent lines.

The CO_2_ assimilation rate was also measured to investigate the impact of AOX alteration on photosynthesis beyond the light phase. WT and *aox* KO plants showed similar CO_2_ assimilation rates, while AOX OE plants showed a significant reduction (Figure 4A). In the latter, in fact, the maximum CO_2_ assimilation rate, recorded after 10 min of light adaptation at saturating light (300 μmol photons m^−2^ sec^−1^), was approximately 35% lower than that of WT (*n* = 4, *P* < 0.05) (Figure 4B). Similar results were obtained in plants grown under high light conditions, with *aox* KO plants indistinguishable from WT, while AOX OE plants showed reduced CO_2_ fixation capacity (Figure S8). These results indicated that the overexpression of *AOX* impacted negatively photosynthetic carbon fixation capacity, aligning with the observed growth reduction.

### AOX plays an essential role when the cytochrome pathway is inhibited

**Figure 5:**
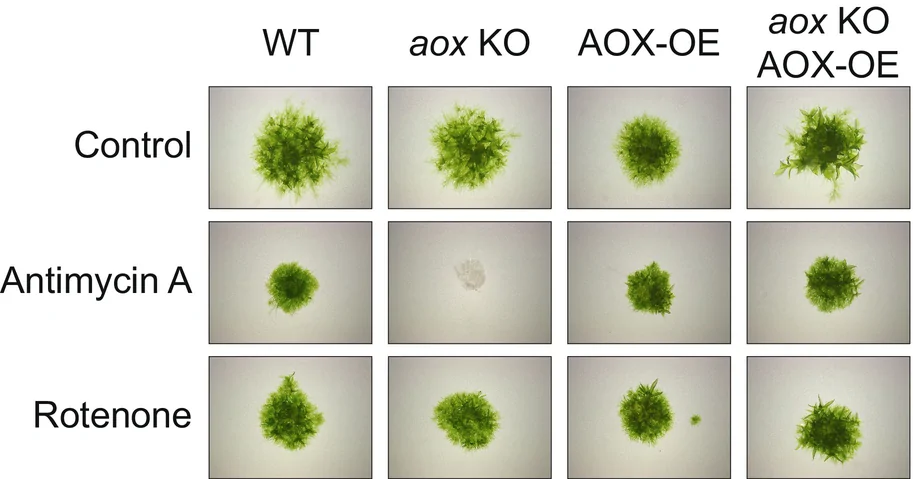
Impact of antimycin A and Rotenone on *Physcomitrium patens* growth. 10 days old protonema of WT, *aox* KO, AOX OE and rescue lines (*aox* KO complemented with AOX OE) with a diameter of 2 mm were transferred to fresh PPNO_3_ medium and cultivated for 21 days without inhibitors (Control) or in the presence of 8 μm antimycin A or 50 μm Rotenone to inhibit the activity of CIII and CI, respectively. Representative images from six independent biological replicates (*n* = 6) are shown, including at least two independent lines for each of the *aox* KO and AOX OE genotypes. Bar = 2 mm.

Taking into account the high complementarity in O_2_ consumption capacity between the cytochrome and AOX pathways, the phenotype of *aox* KO/OE plants was further investigated by treating plants with the CIII inhibitor antimycin A. Both WT and AOX OE plants exhibited approximately 50% growth reduction after the treatment (*n* = 6) (Figure 5). *aox* KO plants were instead much more severely affected and did not survive in the presence of the inhibitor (Figure 5). To confirm that this growth impairment was specifically due to the absence of AOX and not to any secondary effect, *aox* rescue plants (Figure 1) were also tested. These plants recovered their ability to grow in the presence of antimycin A, confirming that AOX activity was indeed essential when the cytochrome pathway was inhibited. The same experiment was repeated using rotenone, a respiratory inhibitor targeting CI (Figure 5). Rotenone also inhibited growth in all genotypes, but they all survived, demonstrating that the lethality of antimycin A in *aox* KO plants was specifically associated with the inhibition of CIII and not a general effect due to disruption of respiration.

### Impairment of mitochondrial electron transport impacts photosynthetic electron transport

**Figure 6:**
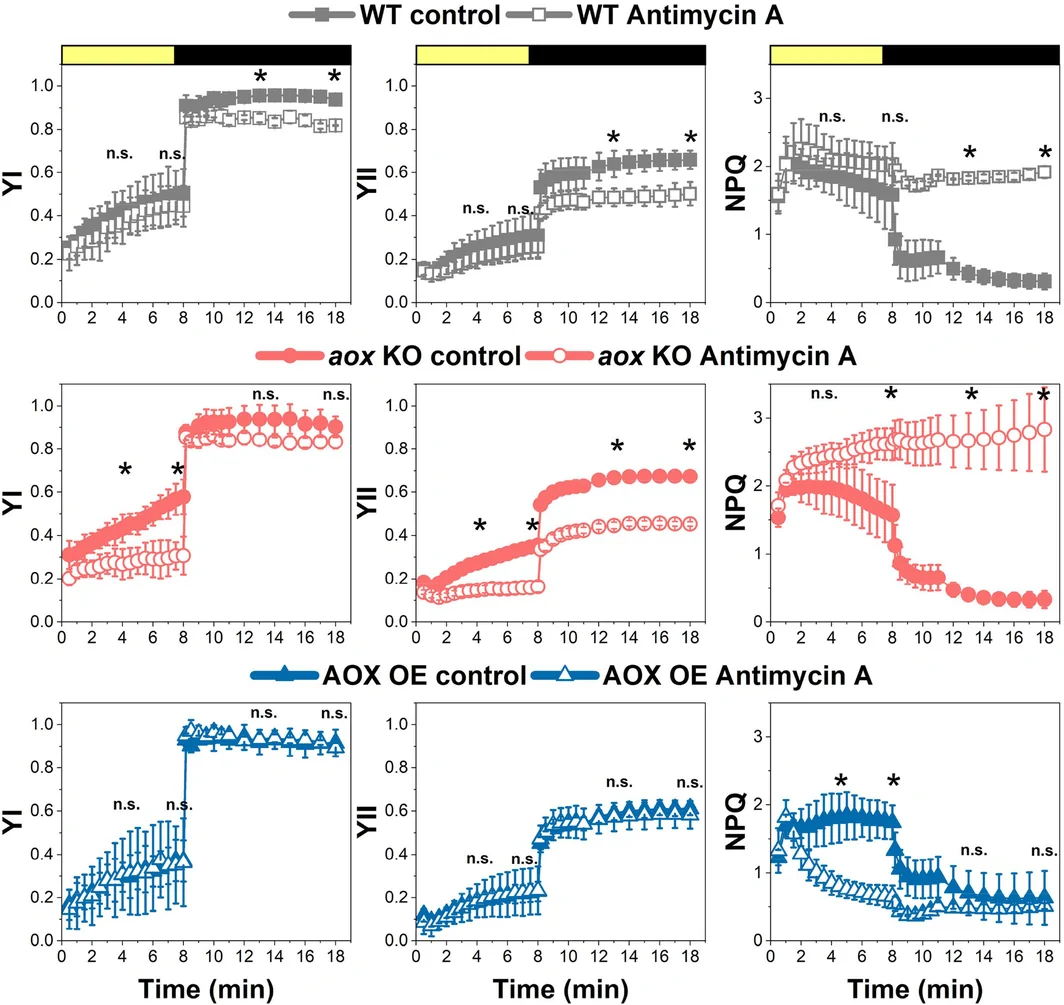
Photosynthetic properties of WT, *aox* KO, AOX OE in *P. patens* protonema treated with antimycin A. Photosynthetic parameters of 10‐day‐old protonema grown in control condition were measured with Dual PAM 100 incubating the tissues with HEPES buffer (control) or 16 μm antimycin A during the 40‐min dark adaptation. During measurements, plants were exposed to 330 μmol photons m^−2^ sec^−1^ actinic light for 8 min (indicated in yellow, top bar) followed by 10‐min darkness (indicated in black, top bar). WT, *aox* KO, and AOX OE plants are shown as black squares, red cycles, and blue triangles, respectively, full or empty for control and samples treated with the inhibitor. Data are shown as average ± SD (*n* = 4). In specific time points (after 4 and 8 min of illumination and 5 and 10 min in the dark) the statistically significant differences are reported (independent sample test, *P < 0.05, n.s.: *P* > 0.05). *aox* KO and AOX OE report pooled data of at least two independent lines.

The above analysis confirms that AOX and the cytochrome pathway are highly complementary, and given the high electron transport capacity of both, the impact of AOX is likely underestimated by the analysis of *aox* KO plants alone. To address this limitation, the photosynthetic properties of *aox* KO and OE plants were re‐assessed following short‐term treatment with antimycin A. In WT plants treated with antimycin A during dark adaptation, Y(I) and Y(II) showed only minor reductions under light, but their recovery in darkness was significantly impaired (Figure 6). The effect was particularly evident on NPQ, suggesting that antimycin A interfered with thylakoid ∆pH relaxation in the dark (Figure 6). The redox state of PQ (1‐qL) and PSI (Y(NA) and Y(ND)) was not impacted under light (Figure S9).

The effects of antimycin A on Y(I) and Y(II) were even more pronounced in *aox* KO plants, which showed larger differences compared to untreated plants and a complete lack of relaxation of NPQ after light exposure (*n* = 4, *P* < 0.05) (Figure 6). In *aox* KO plants, PSI donor‐side limitation was also higher in the light (Figure S9), confirming that the impact on electron transport by antimycin A treatment was generally amplified by the absence of AOX.

Interestingly, in AOX OE plants photosystem efficiency (Figure 6) as well as redox states of PQ and PSI (Figure S9) were indistinguishable from the untreated control, suggesting that the higher AOX activity was able to rescue the effects of CIII inhibition. These results indicated that inhibition of CIII led to alteration in photosynthetic activity and in particular in NPQ relaxation in the dark, effects that were amplified when both cytochrome and alternative pathways were affected, and that were instead rescued by the overexpression of AOX.

In the chloroplast, PGR5‐dependent cyclic electron transport is also an antimycin A sensitive pathway (DalCorso et al., 2008; Munekage et al., 2002), but this is not the case in *P. patens* where the valine responsible for binding to the inhibitor is replaced by lysine (Sugimoto et al., 2013). Our experiment also confirmed this previous result, as antimycin A did not affect the Y(II) and NPQ activation in WT plants (Figure 6).

**Figure 7:**
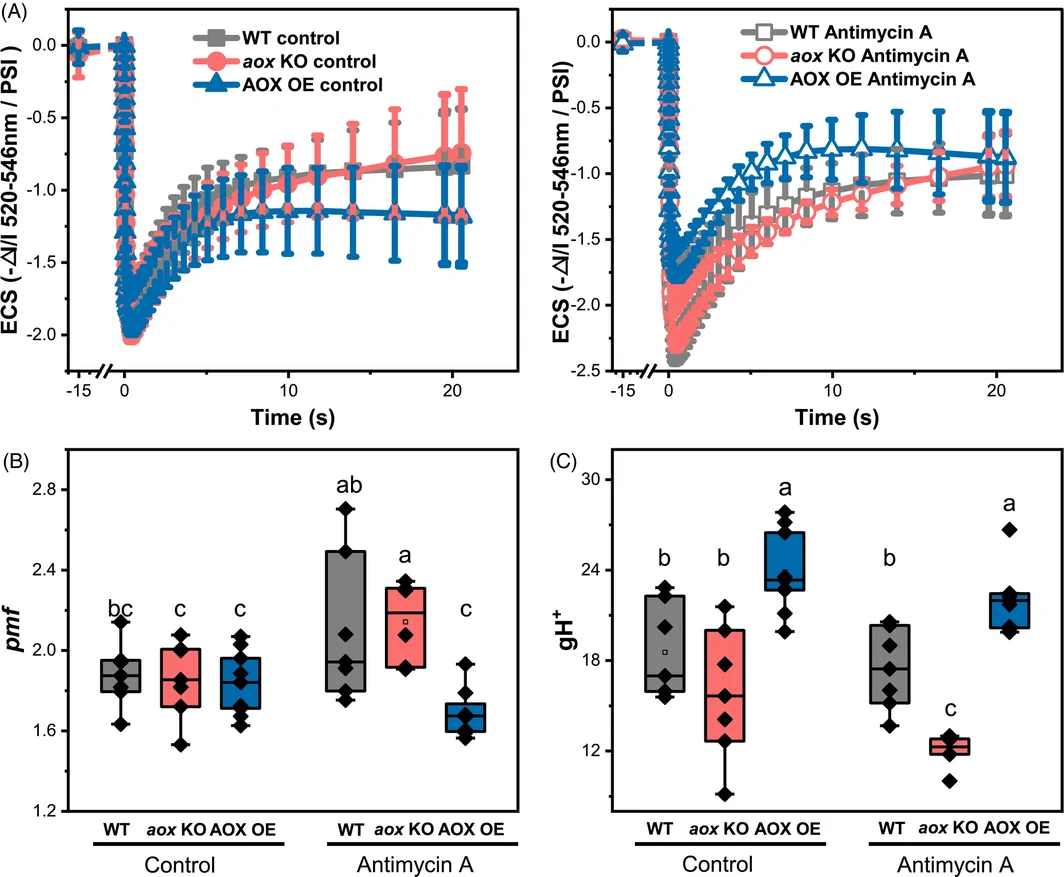
Effect of AOX alteration on *pmf* and proton conductivity (gH^+^) in chloroplast. Proton motive force was estimated by monitoring the carotenoid electrochromic shift. (A) Relaxation electrochromic shift signal in the dark after 400 sec exposition to saturating light (300 μmol photons m^−2^ sec^−1^). Before measurements, samples were incubated in the dark for 40 min with a HEPES buffer (control, left) or with 16 μm antimycin A (right). (B) Proton motive force (*pmf*) size at the end of the illumination. (C) Proton conductivity of protons (gH^+^) across the thylakoid membrane. WT, *aox* KO and AOX OE are shown in black, red and blue, respectively. Average ± SD (*n* ≥ 6) are reported. Different letters indicate statistically significant differences in WT, *aox* KO plants and AOX OE plants, respectively (one‐way anova, *P* < 0.05). *aox* KO and AOX OE report pooled data of at least two independent lines.

As the observed specific effect on the NPQ relaxation points to a possible alteration in the ΔpH across thylakoid membranes, the proton motive force (*pmf*) was assessed in the same plants treated with antimycin A using the ECS relaxation kinetics (Figure 7A). Under normal conditions, AOX alterations had no effects on total *pmf*. However, when the cytochrome pathway was inhibited by antimycin A, *pmf* increased in WT and *aox* KO plants, while it was maintained in AOX OE plants, confirming that AOX can compensate for inhibition of CIII (*n* ≥ 6, *P* < 0.05) (Figure 7B).

The proton conductivity (gH^+^) through the ATPase complex, quantified from the decay of ECS signal in the dark, did not show differences between control and antimycin A‐treated WT (Figure 7C). In contrast, gH^+^ was strongly reduced in *aox* KO plants when the cytochrome pathway was inhibited. AOX overexpression instead enhanced the gH^+^ with respect to WT even in the presence of antimycin A, suggesting that AOX stimulated the proton translocation rate (gH^+^) in the chloroplast (*n* ≥ 6, *P* < 0.05) (Figure 7C). These results indicated that a functional mitochondrial electron transport chain impacts photosynthetic electron transport, particularly affecting chloroplast ATPase activity. Although this effect was not evident in the *aox* KO plant due to the complementary activity of CIII, it became visible when both AOX and CIII were inhibited.

### Modulation of cytosolic and plastidial ATP by mitochondrial respiration during photosynthesis

Previous results suggest that Cytochrome/AOX pathways impact ATP dynamics, especially after the light‐to‐dark transition (Figures 6 and 7). The metabolic consequences of altering these pathways were assessed by generating lines expressing the cytosolic MgATP^2−^‐specific FRET‐based biosensor (ATeam1.03‐nD/nA) in both WT (WT‐cATeam) and *aox* KO (*aox*‐cATeam) backgrounds following an approach already validated in *P. patens* (Vera‐Vives et al., 2024).

**Figure 8:**
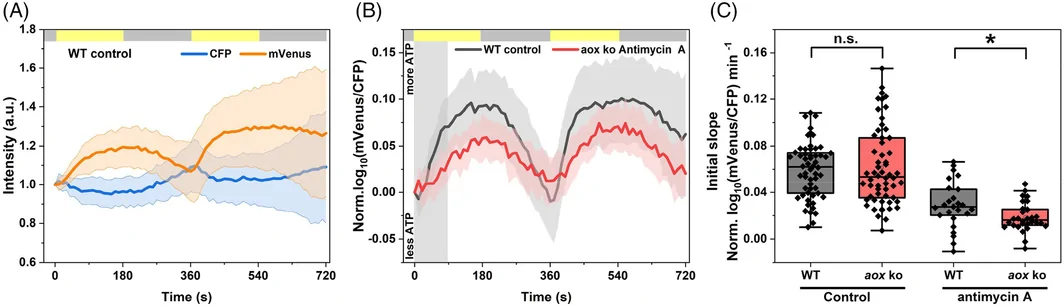
Light‐induced MgATP2^−^ accumulation in the cytosol of *Physcomitrium patens*. Normalised intensities of the single fluorescence channels of WT‐cATeam (A) showing their changes upon dark‐to‐light transitions. Plants were dark‐adapted for 1 h before the measurements, and the first data point was measured before switching light on. Light periods are marked with a yellow bar and correspond to 50 μmol photons m^−2^ sec^−1^ of red light. Fluorescence channels are shown in Orange (mVenus) and cyan (CFP) together with experimental deviation. (B) Normalised log‐transformed FRET ratio during dark‐to‐light transitions of control WT‐cATeam plants (black) and *aox*‐cATeam plants treated with antimycin A (red). (C) Slope during the first 90s of illumination, marked as shadow in the figure (*n* = 56 ROIs from 5 independent samples in WT‐cATeam control; *n* = 58 ROIs from 3 independent samples in *aox*‐cATeam control; *n* = 25 ROIs from 3 independent samples in antimycin A‐treated WT‐cATeam; *n* = 30 ROIs from 3 independent samples in antimycin A‐treated *aox*‐cATeam). The statistically significant differences between WT‐cATeam and *aox*‐cATeam are reported (independent sample test, *P < 0.05, n.s.: *P* > 0.05). WT and *aox* KO report pooled data of at least two independent lines of WT‐cATeam plants and *aox*‐cATeam plants.

After 1 h of dark adaptation to stabilise basal levels of ATP, plants were exposed to two light–dark cycles, 3 min + 3 min each. When the light was turned on, the mVenus signal increased while the CFP signal decreased, indicative of the activation of energy transfer between the two fluorophores (Figure 8A). Indeed, the FRET ratio calculated in WT‐cATeam showed an increase during illumination, followed by a decrease when light was switched off (Figure 8B), indicative of a light‐dependent increase in cytosolic ATP concentration, as previously observed (De Col et al., 2017; Vera‐Vives et al., 2024). The absence of AOX did not alter the basal level of ATP or the response when light was switched on or off (Figures S10 and S11).

Based on all previous results showing the complementarity between CIII and AOX activity, the same experiments were repeated in the presence of a CIII inhibitor (antimycin A). The suppression of CIII in WT‐cATeam caused a slower increase in FRET during the dark‐to‐light transition that did not reach a steady state after 1.5 min as observed in control (Figure S10). However, the impact of antimycin A inhibition on ATP dynamics was more severe in *aox*‐cATeam plants that showed a very small change in fluorescence signals induced by light (Figure S10) and a further reduction in FRET (Figure 8B). The kinetics of ATP accumulation in the light in all different lines were quantified from the kinetics of FRET increase after the light was switched on (*n* = 25–58, *P* < 0.05) (Figure 8C), showing that indeed the rate of ATP synthesis was affected by antimycin A treatment, but the effect was amplified when AOX was also inactivated. These results confirmed the role of mitochondrial respiration in photosynthetic‐dependent ATP dynamics but also showed that AOX contributed to this activity, complementarily to CIII.

**Figure 9:**
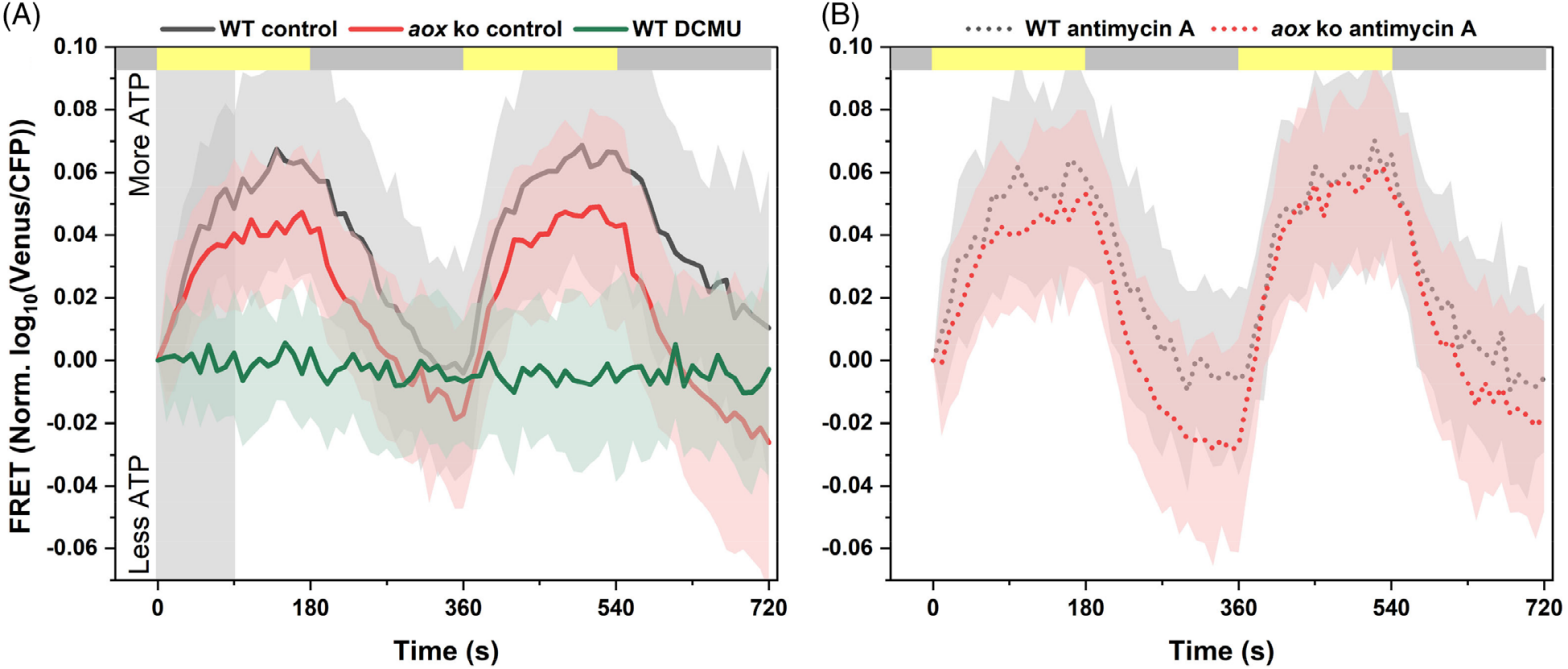
Light‐induced MgATP2^−^ accumulation in the plastid of *Physcomitrium patens*. (A) Normalised log‐transformed FRET ratio during dark‐to‐light transitions of control WT‐pATeam plants (black), DCMU treated WT‐pATeam plants (green) and *aox*‐pATeam plants (red). (B) Normalised log‐transformed FRET ratio during dark‐to‐light transitions of antimycin A‐treated WT‐pATeam plants (black dotted) and *aox*‐pATeam plants (red dotted). *n* = 46 ROIs from 6 independent samples in WT‐pATeam control; *n* = 57 ROIs from 7 independent samples in *aox*‐pATeam control; *n* = 32 ROIs from 3 independent samples in WT‐pATeam DCMU; *n* = 27 ROIs from 4 independent samples in WT‐pATeam antimycin A treated; *n* = 62 ROIs from 5 independent samples in *aox*‐pATeam antimycin A treated. WT and *aox* KO report pooled data of at least two independent lines of WT‐cATeam plants and *aox*‐cATeam plants.

Measurements were also performed with newly generated lines stably expressing the ATeam1.03‐nD/nA in the plastids in both WT (WT‐pATeam) and *aox* KO background (*aox*‐pATeam). When the light was turned on, the plastid‐localised fluorophores showed a clear increase in fluorescence from the acceptor (mVenus) and an opposite trend from the donor (CFP, Figure S12). Consistently, the FRET ratio clearly increased after illumination, reaching a plateau in approximately 60 sec, indicating a fast increase in stromal ATP‐Mg^2+^ concentration (Figure 9A). When the light was turned off, the FRET ratio declined sharply, showing a close dependence on illumination, also during a second light–dark cycle (Figure 9A). The FRET response was completely absent in plants treated with the PSII inhibitor DCMU, suggesting that the observed stromal ATP dynamics are fully dependent on photosynthetic activity (Figure 9A).

As observed for the cytosol, the response of ATP concentration in *aox*‐pATeam, as reflected by the FRET ratio, showed no significant differences compared to WT‐pATeam (Figure 9A). Interestingly, this was also the case when plants were treated with antimycin A even in *aox* KO lines, suggesting that stromal ATP concentrations are not significantly affected by inhibition of respiratory activity, at least on the timescales explored here (Figure 9B). This shows that inactivation of mitochondrial respiration does not directly impact ATP synthesis within the chloroplast.

## DISCUSSION

### AOX activity is highly complementary with cytochrome pathway in *P. patens*

The respiratory O_2_ consumption rate in *P. patens* WT plants showed a limited impact of CIV (KCN) or AOX (SHAM) inhibitors when added alone (Figure 2), but activity was drastically reduced when both inhibitors were applied simultaneously. *aox* KO plants show the same respiratory activity as WT, while a treatment with KCN caused complete inhibition. These results indicate that both the cytochrome pathway and the AOX pathway alone can support full respiratory activity when the other is inhibited, implying the presence of a strong electron transport overcapacity that is normally not fully exploited because of other limiting factors. This result is consistent with previous results showing that in CI or CIV knockout mutants in *P. patens* AOX was able to support strong O_2_ consumption, even higher than in WT (Mellon et al., 2021; Vera‐Vives et al., 2025).

Based on these data, the maximum capacity of the AOX pathway in *P. patens* can be estimated to account for 73% of the total respiratory O_2_ consumption, while it is 84% for the cytochrome pathway. This value represents the maximum AOX capacity measured under conditions where the cytochrome pathway is chemically inhibited, reflecting the potential electron flux through AOX and not its contribution to respiration under steady‐state conditions. AOX capacity has been estimated to be 40–50% in Arabidopsis, tomato and pea (Dinakar et al., 2016; Kühn et al., 2014; Zhu et al., 2018), while a 74% capacity was also reported for *Nicotiana benthamiana* (Lee et al., 2011). In the green alga *Chlamydomonas reinhardtii*, the AOX capacity was estimated to be approx. 50% of the total respiration rate (Mathy et al., 2010), a value thus similar to most vascular plants. These data suggest that AOX capacity is a variable feature in different species and conditions, and it may represent a species‐specific and environment‐sensitive trait rather than following a clear evolutionary trend.

The high electron transport capacity of AOX can explain the AOX OE growth penalty, which is also correlated with a reduced CO_2_ fixation rate (Figures 3 and 4). Excess AOX protein accumulation and activity could cause wasteful dissipation of reducing power under non‐stress conditions, even though this is not evident from oxygen consumption rates. However, there are other possible hypotheses: as AOX is also involved in mitochondrial and chloroplast retrograde signalling pathways, responding to multiple regulators, such as ANAC017‐dependent and ABI4‐dependent pathways (León et al., 2013; Merendino et al., 2020; Ng et al., 2013), the phenotype in AOX OE plants could thus also be associated with a mis‐regulation of these signalling pathways leading to growth alterations.

The moss *P. patens* possesses only a single copy of the *AOX* gene, lacking the gene redundancy found in many vascular plants (Neimanis et al., 2013). This feature enables us to assess the AOX functional role without compensation among paralogs. Despite the high electron transport capacity of the alternative pathway, *P. patens aox* KO plants did not show any significant differences in growth and photosynthetic parameters compared to WT (Figures 3 and 4), not even by testing different tissues, including gametophores where AOX is more abundant or under various abiotic stress conditions (Figures S3 and S7). While it is not possible to exclude that AOX absence could be impactful in other conditions not assessed here, nonetheless these data suggest that *P. patens aox* KO plants could cope well with several different stresses.

The weak impact of the absence of AOX can be explained by considering that the cytochrome pathway is capable of supporting enough electron transport activity to compensate, thus minimising the negative repercussions. This strong complementarity emerges clearly from the treatment with the CIII inhibitor antimycin A, where the activity of AOX becomes essential, leading to a lethal phenotype in *aox* KO plants (Figure 5). In Arabidopsis, which carries multiple AOX isoforms, antimycin A treatment of *aox1a* knockout mutants caused effects such as inhibition of photosynthesis and increased ROS compared to WT (Strodtkötter et al., 2009) and more severe inhibition of root elongation (Fuchs et al., 2022). However, *aox1a* KO were still able to survive, likely because of the presence of other AOX isoforms. The complete lethality observed in the *P. patens aox* KO plant upon CIII inhibition, and its full rescue by AOX complementation (Figure 5), provide new and solid *in vivo* evidence for the essential complementarity between the cytochrome pathway and AOX. This complementarity is also visible in the opposite direction. In WT plants where CIII role is inhibited with antimycin A, the impact on photosynthetic properties is rescued by AOX overexpression (Figure 6).

The available literature points to a specific role for AOX especially under stress conditions, dissipating excess reducing power and decreasing ROS formation (Arnholdt‐Schmitt et al., 2006; Giraud et al., 2008; Vanlerberghe, 2013). The data shown here reveal that the cytochrome pathway also plays an impactful role under different environmental conditions. This suggests that the mitochondrial respiratory activity itself by consuming reducing power produced by photosynthesis contributes to minimise the over‐reduction in stressing conditions, independently from the complex catalysing the reaction. Rather than having a specific role in the response to stress, AOX supports the essential role of mitochondrial electron transport, providing flexibility and robustness.

### Mitochondrial respiration is essential for efficient photosynthetic electron transport

Based on results supporting the essentiality of respiration in photosynthetic cell metabolism, the analysis of antimycin A‐treated *aox* KO represents a unique tool for investigating, at least in the short term, the effects of completely blocking mETC by inactivating all major mechanisms for ubiquinol re‐oxidation. While in the long term this inactivation is lethal (Figure 5), a short‐term treatment allows assessing the role of mitochondrial respiration in a photosynthetically active cell.

Interestingly, short‐term incubation with antimycin A did not affect the ability of the plastid to synthesise ATP (Figure 9), consistent with the idea that the chloroplast is capable of converting light into chemical energy even when respiration is inactivated, as also observed in mutants mutated in CIV and V (Vera‐Vives et al., 2024, 2025). Blocking the mitochondrial electron transport, however, still affects the photosynthetic apparatus activity. After a saturating illumination is switched off, in fact, the proton conductivity (gH^+^) of chloroplast ATPase is reduced (Figure 7) and a larger ΔpH across the membrane of thylakoids is maintained. This effect is stronger when plants are exposed to intense illumination and is much smaller if exposed to growing light, suggesting that chloroplasts are still capable of synthesising ATP, but the capacity is more easily saturated (Figure S13).

Chloroplast ATPase is regulated by its redox state, but it should be fully active under illumination in the conditions tested here (Sekiguchi et al., 2022, 2024). The most likely explanation for the alterations observed under saturating illumination is thus that ATPase is substrate limited due to the so‐called metabolic control (Geigenberger et al., 2010). If respiration is blocked, the metabolic activity of the cell beyond the chloroplast is likely reduced, impairing the consumption of triose phosphates exported from the chloroplast and consequently the regeneration of Pi (Raghavendra & Padmasree, 2003; Taniguchi & Miyake, 2012). When exposed to dim light, the chloroplasts are still able to synthesise and consume ATP (Figure 9), but under saturating light the respiration and the rest of the cell metabolism are essential to consume the products of photosynthesis with a rate sufficient to support efficient electron transport.

### Respiration is essential for ATP supply in the cytosol, also with a contribution from AOX

Plants are autotrophic organisms where photosynthesis is the ultimate source of energy, but the chloroplasts have a limited capacity to export ATP to the cytosol, whose supply is instead strongly dependent on mitochondria and respiration (Shameer et al., 2019; Vera‐Vives et al., 2024) (Figure 8). The stromal reductant exported from the chloroplast using redox shuttles such as the malate valve or the TP/3‐phosphoglycerate shuttle (Shameer et al., 2019; Taniguchi & Miyake, 2012; Voon et al., 2018) is exploited mainly by the cytochrome pathway to produce ATP to be supplied to the cytosol and the rest of the cell (Figure 8) (Vanlerberghe et al., 2020; Vera‐Vives et al., 2024). Other pathways, such as glycolysis and the TCA cycle, also contribute to ATP accumulation (O'Leary et al., 2019), but the results shown here, when both the cytochrome and alternative pathways were inhibited, confirm that active mETC provides an essential contribution to the supply of ATP (Figure 8), even under fully autotrophic conditions.

In this context, it is worth considering what the role of AOX would be, considering that its activity can be largely compensated for by the cytochrome pathway. When the demand for ATP is low, as in stress conditions, the activity of CV and therefore the dissipation of the proton motive force across the inner mitochondrial membrane will also be slowed down. Under these conditions, electron flow through proton pumping CIII and CIV becomes restricted by an increase in ∆pH, in a mechanism called ‘adenylate control’ that is a major regulator of electron flow through the cytochrome pathway (Igamberdiev & Kleczkowski, 2006; O'Leary et al., 2019). In these conditions, however, the mitochondrial ability to consume reducing power would also be limited, possibly leading to excess reducing power and over saturation of the photosynthetic ETC. AOX, however, is not constrained by the availability of ADP, which enables the cell to maintain high rates of electron flow to oxygen and continuous consumption of reducing equivalents, even under energy‐limiting conditions (Chadee et al., 2021; Vanlerberghe & McIntosh, 1997). Thus, AOX activity maintains the mitochondrial electron transport capacity and thus its ability to consume reducing power even when metabolic demand is limited by external factors. This enables mitochondria to act as an electron sink for reductants produced in the chloroplast, decoupling reducing power consumption from ATP production in conditions of low demand (Vanlerberghe et al., 2020). Although the major impact on cell metabolism is exerted by mitochondrial electron transport itself, AOX provides robustness and flexibility to enable its activity under a wide range of environmental conditions.

## MATERIALS AND METHODS

### Plant materials and growth condition

Protonemal tissue of *P. patens* WT (Gransden), *aox* KO plants and AOX OE plants was grown on minimum PPNO_3_ medium in a growth chamber at 24°C, 50 μmol photons m^−2^ sec^−1^ of light (control light in the text) and set in a long photoperiod (16 h light/8 h dark). Physiological and biochemical experiments were performed on 10‐day‐old protonema.

### Plant phenotyping

Growth rate assays were started with fresh protonema, after 10 days of growth, 2 mm diameter protonema were transferred to new PPNO_3_ medium plates or PPNO_3_ medium containing 8 μm antimycin A or 50 μm Rotenone and grew at 50 μmol photons m^−2^ sec^−1^ for 21 days, the plants for high light stress assessment were grown at 500 μmol photons m^−2^ sec^−1^. Inhibitor concentrations were previously validated in *P. patens* tissues (Mellon et al., 2021; Vera‐Vives et al., 2025). To assess salt and osmotic stress, 2 mm diameter 10‐day‐old protonema were grown on PPNO_3_ medium with a filter for 2 weeks; then, the filters with moss colonies were transferred to PPNO_3_ medium containing 0.4 m NaCl and 0.8 m sorbitol for 1 week, respectively.

The colony size was quantified as previously described (Storti et al., 2019). The plates containing with colonies were scanned with a Konica Minolta Bizhub C280 scanner in high resolution (600 ppi). Images were processed with FIJI (https://fiji.sc/) using the ‘threshold colour’ plugin to remove the background. Then, integrated density (area × mean density) was measured for each colony and normalised by each initial area to characterise the 3D growth of gametophore. The maximum PSII efficiency (*Fv/Fm*) was detected using PAM imaging during growth.

### Moss transformation and line selection

Selected upstream and downstream homologous recombination regions from *PpAOX* gene were amplified by PCR from WT gDNA and cloned, respectively, into BHRf and BNRf (Gerotto et al., 2016) plasmids for *aox* knockout lines. Full‐length coding sequence of *PpAOX* was amplified from WT cDNA with the PpAOX‐attB1/2 primers and cloned into pT1OG (Aoyama et al., 2012) plasmid with PpEF1‐α promoter for AOX overexpression lines. Protoplast transformation and line screening were performed as previously described (Gerotto et al., 2016). RNA was afterward purified with RNeasy Plant Mini Kit (Vazyme) and used as a template for cDNA synthesis with RevertAid Reverse Transcriptase (Thermo Scientific) to verify *aox* gene expression in WT, *aox* KO and AOX OE plants.

New ATP reporter lines were generated in both the WT and *aox* KO backgrounds using cytATeam_ pT1OG and plastidATeam_ pT1OG expression vectors with the coding sequence of ATeam1.03‐nD/nA (Vera‐Vives et al., 2024; Zheng et al., 2025). The isolated colonies, expressing the ATeam probe, were first checked with a Leica DMI6000 microscope. If mVenus fluorescence was observed, they were then further examined under a Leica STELLARIS 8 confocal microscope to further check the probe localisation and function.

### Immunoblot analysis

Protonema and gametophore were ground in liquid nitrogen, and total proteins were extracted in sample buffer (50 mm TRIS pH 6.8, 100 mm DTT, 2% (w/v) SDS and 10% (w/v) glycerol). Total protein extractions were quantified by chlorophyll content and then loaded to every well at different dilution factors. After SDS‐PAGE, proteins were transferred to a PVDF membrane (Millipore). Membranes were blocked with 5% milk then probed with specific primary antibodies: anti‐AOX1/2, Agrisera, catalogue number AS04054; custom‐made anti‐NAD9 (CI); custom‐made anti‐SDH1‐1 (CII), anti‐a‐MPP (CIII), anti‐COX2 (CIV), anti‐b subunit (CV) and anti‐AtUCP1.

### Oxygen consumption measurement

Measurements of oxygen consumption (respirometry) were performed as in (Vera‐Vives et al., 2025), with 10‐day‐old intact 1 cm^2^ protonema using the NextGen‐O_2_k and the PhotoBiology (PB)‐Module (Oroboros Instruments, Innsbruck). Briefly, protonema were placed on the sample holder in a 2 ml chamber with sterile PPNO_3_ medium containing 10 mm NaHCO_3_ after 40‐min dark adaptation. The oxygen concentration was monitored for 10 min in the dark to assess the total respiration rate. KCN or SHAM (at a final concentration of 1 mm, as validated in previous studies (Mellon et al., 2021; Vera‐Vives et al., 2025)) was added to the chamber using Hamilton syringes. The oxygen concentration was monitored for another 10 min in the dark to assess alternative or cytochrome respiration rate. All the data were normalised by the chlorophyll content in each sample.

### Spectroscopic analysis

A Dual PAM‐100 (Heinz Walz, Effeltrich, Germany) was used to measure PSI and PSII parameters at room temperature simultaneously. In photosynthetic phenotype measurement, after 40‐min dark adaptation, the maximum fluorescence and the maximum change in P700 were measured using a saturating pulse. Afterward, 10‐day‐old protonema or 2‐month‐old gametophore without rhizoid were illuminated at 63 or 330 μmol photons m^−2^ sec^−1^ for 8 min followed by 5‐min darkness. In short‐term antimycin A measurement, 10‐day‐old protonema grown with PPNO_3_ medium were submerged in H_2_O or 16 μm antimycin A during 40‐min dark adaptation. Then, protonema were exposed to 330 μmol photons m^−2^ sec^−1^ for 8 min followed by 10‐min darkness. PSI and PSII parameters were calculated as follows: Y(I) as (Pm′ − P)/Pm, Y(ND) as P/Pm, Y(NA) as (Pm − Pm′)/Pm. Pm refers to the maximal level of P700+ (oxidised P700) in the dark by application of an SP in the presence of far‐red light (720 nm). Pm′ refers to the maximal level of oxidised P700 during actinic light illumination which was determined by SP application. P refers to the P700 signal recorded just before an SP. Y(II) as (Fm′ − Fs)/Fm′, NPQ as (Fm − Fm′)/Fm′, qL as (Fm′ − F)/(Fm′ − Fo) × Fo′/F, where Fm and Fo represent the maximum and minimum fluorescence yields. Y(I), quantum yield of PSI photochemistry; Y(ND), quantum yield of non‐photochemical quenching due to PSI donor‐side limitation; Y(NA), quantum yield of non‐photochemical quenching due to PSI acceptor side limitation; Y(II), effective quantum yield of PSII photochemistry; NPQ, non‐photochemical quenching in PSII.

Electro Chromic Shift (ECS) spectra were recorded with a JTS‐10 system (Biologic) as described in (Gerotto et al., 2016). Before test, protonema were imbibed with HEPES buffer (20 mm HEPES, pH 7.5 and 10 mm KCl) or 16 μm antimycin A during 40‐min dark acclimation. For each measure, the background signal at 546 nm was subtracted from the 520 nm signal to eliminate the contribution of scattering and cytochromes. Single turnover flash spectroscopy was performed to obtain the total number of active reaction centres in the chloroplasts. Protonema were exposed to 350 μmol photons m^−2^ sec^−1^ for 6 min, the relaxation kinetics were recorded after light was switched off. The *pmf* was calculated as the difference between the maximum signal at the light steady state and the minimum level of ECS in the dark. ATPase activity in chloroplast was accessed by fitting the first 300 ms of the ECS decay curve with a first order exponential decay kinetic and indicated as the inverse of the decay time constant (Avenson et al., 2005).

### Gas exchange measurement

The CO_2_ assimilation rate was measured with the LI‐6800 portable photosynthetic system (Li‐cor Biosciences, Lincoln, NE, USA) on 8‐week‐old gametophytes of *P. patens*. Gametophytes were cultivated on the PPNO_3_ medium with a cellophane filter to separate them more easily from the medium after measurement to obtain accurate dry weight. Gametophytes with medium block were placed in the bryophyte chamber (part no.: 6800‐24) with the large light source (part no.: 6800‐03). All measurements were made at an air humidity of 80–85%, CO_2_ concentration of 400 ppm, and chamber temperature of 25 °C. After gas exchange measurements, the CO_2_ assimilation rate was recalculated by dry weight. The light response curve was performed after 10‐min light induction at 300 μmol photons m^−2^ sec^−1^. For each light intensity (600, 400, 250, 150, 100, 50, 20, 0 μmol photons m^−2^ sec^−1^), the CO_2_ assimilation rate was logged upon reaching a steady condition after 2 min.

### Subcellular protein localisation

The *PpAOX* coding region was cloned into the PT1OGY plasmid. 5‐ or 6‐day‐old protonemal tissue grown on PPNH_4_ medium was collected for protoplast generation. PEG‐mediated transformation was performed as previously described (Alboresi et al., 2010). The transfected protoplasts were incubated in the dark at 23°C for 24–72 h before taking images. YFP signals and chlorophyll auto‐fluorescent signals were visualised under the ZEISS LSM900 confocal microscope. The YFP signals were excited using 488 nm and detected at 505–576 nm for detection of YFP. The chloroplasts auto‐fluorescence was taken with excitation at 640 nm and emission at 600–700 nm. MitoTracker Orange CMTMRos was used to confirm mitochondrial localisation.

### Confocal imaging

A piece of 10‐day‐old protonema was placed on a slide with a drop of liquid PpNO_3_ medium with 10 mm NaHCO_3_, which was necessary to supply CO_2_ for photosynthesis throughout the experiment, then covered with a coverslip. The coverslip was then fixed with 3 m tape to avoid desiccation of the sample and keep air exchange during the measurement. Then, the samples were submerged in medium with or without inhibitors during 1‐h dark adaptation. DCMU, an inhibitor of PSII, was used at a concentration of 500 μm; antimycin A, an inhibitor of CIII of mitochondrial electron transport chain, was applied at 16 μm. Samples were illuminated using a red‐filtered (580–630 nm) light (halogen lamp connected to an optic fibre). Light intensity was set to 50 μmol photons m^−2^ sec^−1^ using a light meter (LI‐250A; Li‐Cor, Lincoln, NE, USA) before measurement.

Imaging was performed using a 20×/0.75 dry objective using a Leica STELLARIS 8 confocal microscope (Leica Microsystems, Wetzlar, Germany). An Argon laser (455 nm) was used to excite both CFP and chlorophylls, with laser power set at 12.5%. A scan was performed every 10 sec. The first 3 scans were performed in darkness, and the average was used to quantify the basal ATP levels. Light was then turned on and off every 3 min. Three emission channels were set up: CFP (464–506 nm), mVenus (520–561 nm) and auto‐fluorescence of Chls (670–690 nm). The acquired fluorescence datasets were analysed using the MATLAB‐based Redox Ratio Analysis software (RRA) (Fricker, 2016) as described in (Vera‐Vives et al., 2024). The number of ROIs is indicated in the figure legends.

## AUTHOR CONTRIBUTIONS

S‐LT contributed to all data generation and analysis. AMV‐V, HW and CB contributed to the O_2_ consumption measurement, lines generation and growth characterisation. XH contributed to CO_2_ assimilation and JTS measurement. TM and AA contributed to the data analysis and supervision. S‐LT and TM contributed to the writing – original draft. AA, XH, HW, CB and AMV‐V contributed to the review and editing.

## CONFLICT OF INTEREST

None declared.

## Supporting information


**Figure S1.** Subcellular location of PpAOX‐YFP in moss *P. patens* protoplast. PpAOX‐YFP was transformed in *P. patens* protoplasts and YFP signal was detected under ZEISS LSM900 confocal microscope. Bars = 5 μm.
**Figure S2.** Isolation of *aox* KO and AOX OE lines in *P. patens*. (A) Scheme for representation of *aox* KO event. Blue boxes represent 5′ and 3′UTR, green boxes represent exons, black continuous lines introns and black dashed lines adjacent non‐coding regions, grey box represents hygromycin resistance cassette. Homology regions were indicated by brackets. (B) AOX expression in WT, *aox* KO and AOX OE lines. *PpAOX* gene expression was assessed by RT‐PCR in WT, two independent *aox* KO, two independent AOX OE lines and one *aox* rescue line protonema grown under control light. Expression of Actin 2 was also reported as reference.
**Figure S3.** Salt and osmotic stress growth phenotypes of *P. patens* WT, *aox* KO lines and AOX OE lines. 10‐day‐old protonema colonies with a diameter of about 2 mm were transferred and grown under control light (50 μmol photons m^−2^ sec^−1^) on PPNO_3_ medium supplemented 0.4 m NaCl or 0.8 m Sorbitol, respectively. Photographs were taken and PSII maximum efficiency (*Fv/Fm*) was measured after 21‐day growth. Bar = 2 mm. Data are shown as average ± SD (*n* = 6). The asterisk indicates statistically significant differences (independent sample test, *P* < 0.05).
**Figure S4.** PSI photosynthetic characteristics of WT, *aox* KO, AOX OE protonema at different light intensities. 10‐day‐old protonema grown under control light (50 μmol photons m^−2^ sec^−1^) were measured with Dual PAM 100. After 40 min dark adaptation, plants were exposed to 63 μmol photons m^−2^ sec^−1^ or 330 μmol photons m^−2^ sec^−1^ actinic light for 8 min (indicated in yellow, top bar) followed by 5 min darkness (indicated in black, top bar). WT, *aox* KO and AOX OE are shown in black square, red cycle, and blue triangle, respectively. Data are shown as average ± SD (*n* = 4). The n.s. indicate statistically no significant differences of three plants (one‐way anova, *P* > 0.05) after 4, and 8 min of illumination and after 2 and 5 min in the dark. *aox* KO and AOX OE report pooled data of at least two independent lines.
**Figure S5.** PSII photosynthetic characteristics of WT, *aox* KO, AOX OE protonema at different light intensities. 10‐day‐old protonema grown under control light (50 μmol photons m^−2^ sec^−1^) were measured with Dual PAM 100. After 40 min dark adaptation, plants were exposed to 63 μmol photons m^−2^ sec^−1^ or 330 μmol photons m^−2^ sec^−1^ actinic light for 8 min (indicated in yellow, top bar) followed by 5 min darkness (indicated in black, top bar). WT, *aox* KO, AOX OE are shown in black square, red cycle, and blue triangle, respectively. Data are shown as average ± SD (*n* = 4). The n.s. indicate statistically no significant differences of three plants (one‐way anova, *P* > 0.05) after 4, and 8 min of illumination and after 2 and 5 min in the dark. *aox* KO and AOX OE report pooled data of at least two independent lines.
**Figure S6.** AOX expression in different developmental stages of *P. patens*. (A) AOX gene expression map describing the transcript expression patterns of AOX during development in *P. patens* Gransden and Reute ecotypes. The graph was recomposed from PEAT moss https://peatmoss.plantcode.cup.uni‐freiburg.de/. (B) Transcript expression of the AOX in protonema and gametophore in *P. patens* Gransden. Data was downloaded from PEAT moss. Data are shown as average ± SD (*n* = 3). The asterisk indicates statistically significant differences (independent sample test, *P* < 0.01). (C) The amounts of protein on respiratory electron transport chain in protonema and gametophore. 10‐day‐old protonema and 2‐month‐old gametophore were grown on PPNO_3_ medium under control light. Total protein extraction were loaded, 1× loading corresponded to 2 μg of total chlorophyll.
**Figure S7.** Photosynthetic fluorescence parameters in *P. patens* gametophores. Gametophores were cultivated on PPNO_3_ medium at control light (50 μmol photons m^−2^ sec^−1^). 30‐day‐old gametophores were cut and measured at 63 μmol photons m^−2^ sec^−1^ or 330 μmol photons m^−2^ sec^−1^. WT and *aox* KO lines are shown in dark green squares and light green cycles, respectively. Average ± SD (*n* = 4) are reported. *aox* KO and AOX OE report pooled data of at least two independent lines.
**Figure S8.** CO_2_ fixation capacity of *Physcomitrium patens* grown under high light. (A) Light response curve of CO_2_ assimilation rate. (B) Steady‐state CO_2_ assimilation rate at saturated light intensity (300 μmol photons m^−2^ sec^−1^). Gametophores were cultivated on PPNO_3_ medium at high light (500 μmol photons m^−2^ sec^−1^). CO_2_ assimilation rate was measured after 30 days of growth. WT, *aox* KO, and AOX OE lines are shown in black, red, and blue, respectively. Average ± SD (*n* = 4) are reported. The different letters indicate statistically significant differences in WT, *aox* KO plants and AOX OE plants, respectively (one‐way anova, *P* < 0.05). *aox* KO and AOX OE report pooled data of at least two independent lines.
**Figure S9.** PQ and PSI redox state of WT, *aox* KO, AOX OE lines in *P. patens* protonema treated with antimycin A. 10‐day‐old protonema grown at control light (50 μmol photons m^−2^ sec^−1^) and were measured with Dual PAM 100. Plants were submerged with HEPES buffer (control) or 16 μm antimycin A during the 40 min dark adaptation, then plants were exposed to 330 μmol photons m^−2^ sec^−1^ actinic light for 8 min (indicated in yellow, top bar) followed by 10 min darkness (indicated in black, top bar). WT, *aox* KO, and AOX OE plants are shown in black square, red cycle, and blue triangle respectively. Data are shown as average ± SD (*n* = 4). The different symbols indicate statistically significant differences of the three plants (one‐way anova, *P < 0.05, n.s.: *P* > 0.05) after 4, and 8 min of illumination and after 5 and 10 min in the dark. *aox* KO and AOX OE report pooled data of at least two independent lines.
**Figure S10.** The contribution of complex III and AOX to light‐induced MgATP^2−^ accumulation in the cytosol of *Physcomitrium patens*. Normalized intensities of the single fluorescence channels of *aox*‐cATeam plants control (A), WT‐cATeam (B) *aox*‐cATeam plants (C) treated with 16 μm antimycin A, showing their changes upon dark‐to‐light transitions. Plants were dark‐adapted for 1 h before the measurements, and the first data point was measured before switching light on. Light periods are marked with a yellow bar and correspond to 50 μmol photons m^−2^ sec^−1^ of red light. Fluorescence channels are shown in Orange (mVenus) and cyan (CFP) together with experimental deviation (*n* = 58 ROIs from 3 independent samples in *aox*‐cATeam control; *n* = 25 ROIs from 3 independent samples in antimycin A treated WT‐cATeam; *n* = 30 ROIs from 3 independent samples in antimycin A treated *aox*‐cATeam).(D) Normalized log‐transformed FRET ratio during dark‐to‐light transitions of WT‐cATeam plants treated with antimycin A (red) and *aox*‐cATeam plants (black). WT and *aox* KO report pooled data of at least two independent lines of WT‐cATeam plants and *aox*‐cATeam plants.
**Figure S11.** Comparison of unnormalized chloroplast stroma and cytosolic FRET ratio at basal level. The data was the average of three points measured before switching light on. The different letters indicate statistically significant differences in WT plants and *aox* ko plants, respectively (one‐way anova, *P* < 0.05). WT and *aox* KO report pooled data of at least two independent lines of WT‐cATeam plants and *aox*‐cATeam plants.
**Figure S12.** Single channels dynamics in the plastid of *Physcomitrium patens*.
**Figure S13.** Photosynthetic propoters at growth light in *P. patens* protonema treated with antimycin A.

## Data Availability

The data supporting the findings of this study are available within the article and its Supporting Information.
