# Supplementary material for: Essential mitochondrial activity in Physcomitrium patens relies on complementary cytochrome and alternative oxidase pathways
Source: Plant J. 2026 Sep 17;127(5):e71127. doi: 10.1111/tpj.71127 (PMC13584256; doi:10.1111/tpj.71127)
Supplement: Supplementary file 1 [file TPJ-127-0-s001.pdf]

SUPPORTING INFORMATION

Essential Mitochondrial Activity in *Physcomitrium patens* Relies on Complementary Cytochrome and Alternative Oxidase Pathways

Shun-Ling Tan, Antoni M Vera-Vives, Heyu Wang, Xing Huang, Claudia Beraldo, Alessandro Alboresi, Tomas Morosinotto

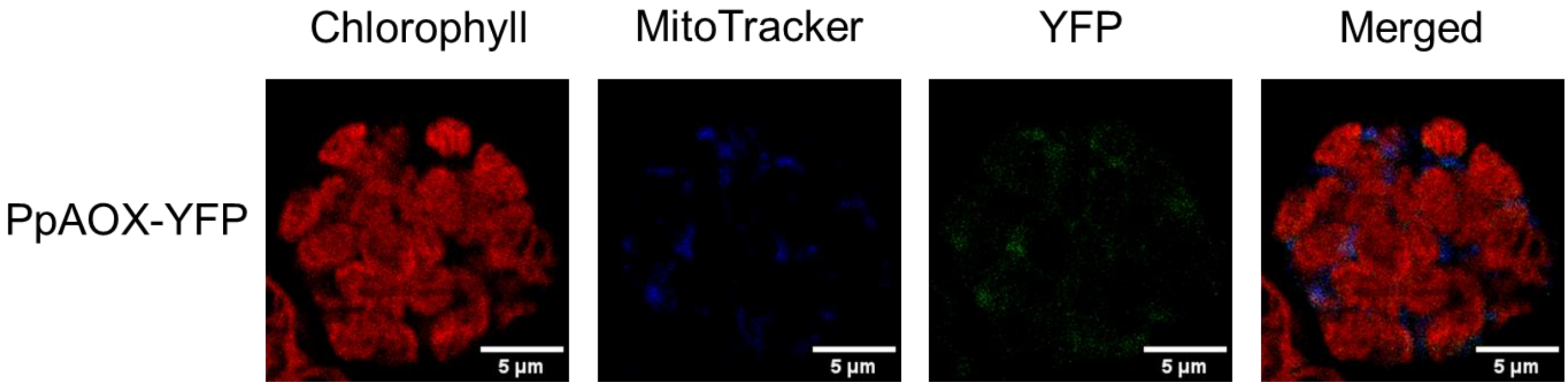

Figure S1 Subcellular location of PpAOX-YFP in moss *P.patens* protoplast. PpAOX-YFP was transformed in P. patens protoplasts and YFP signal was detected under ZEISS LSM900 confocal microscope. MitoTracker was used to stain mitochondria. Bars = 5 um.

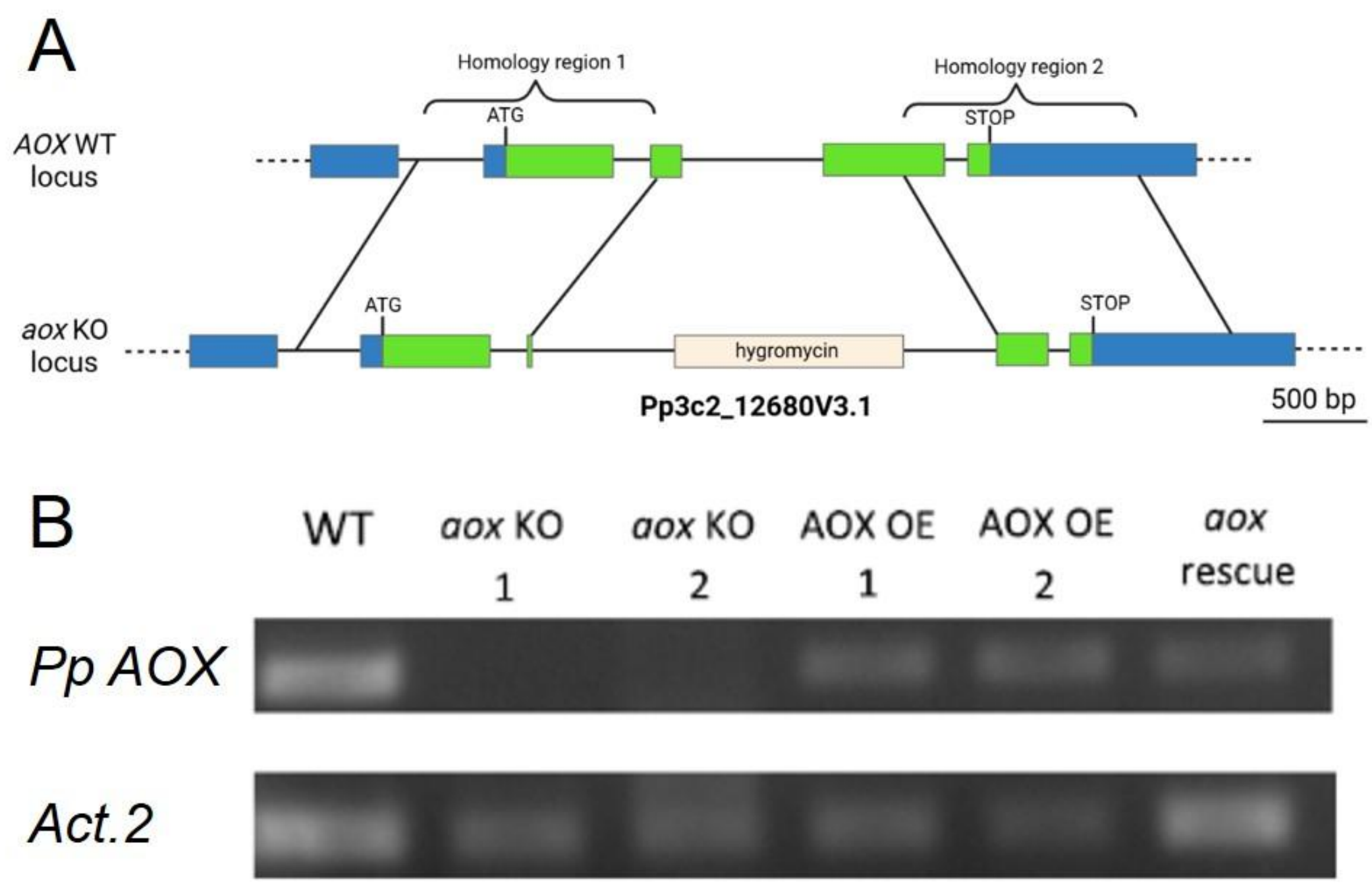

Figure S2. Isolation of *aox* KO and AOX OE lines in *P. patens*. A, Scheme for representation of *aox* KO event. Blue boxes represent 5' and 3'UTR, green boxes represent exons, black continued lines introns and black dashed lines adjacent non-coding regions, grey box represents hygromycin resistance cassette. Homology regions were indicated by brackets. B. AOX expression in WT, *aox* KO and AOX OE lines. *PpAOX* gene expression was assessed by RT-PCR in WT, two independent *aox* KO, two independent AOX OE lines and one *aox* rescue line protonema grown under control light. Expression of Actin 2 was also reported as reference.

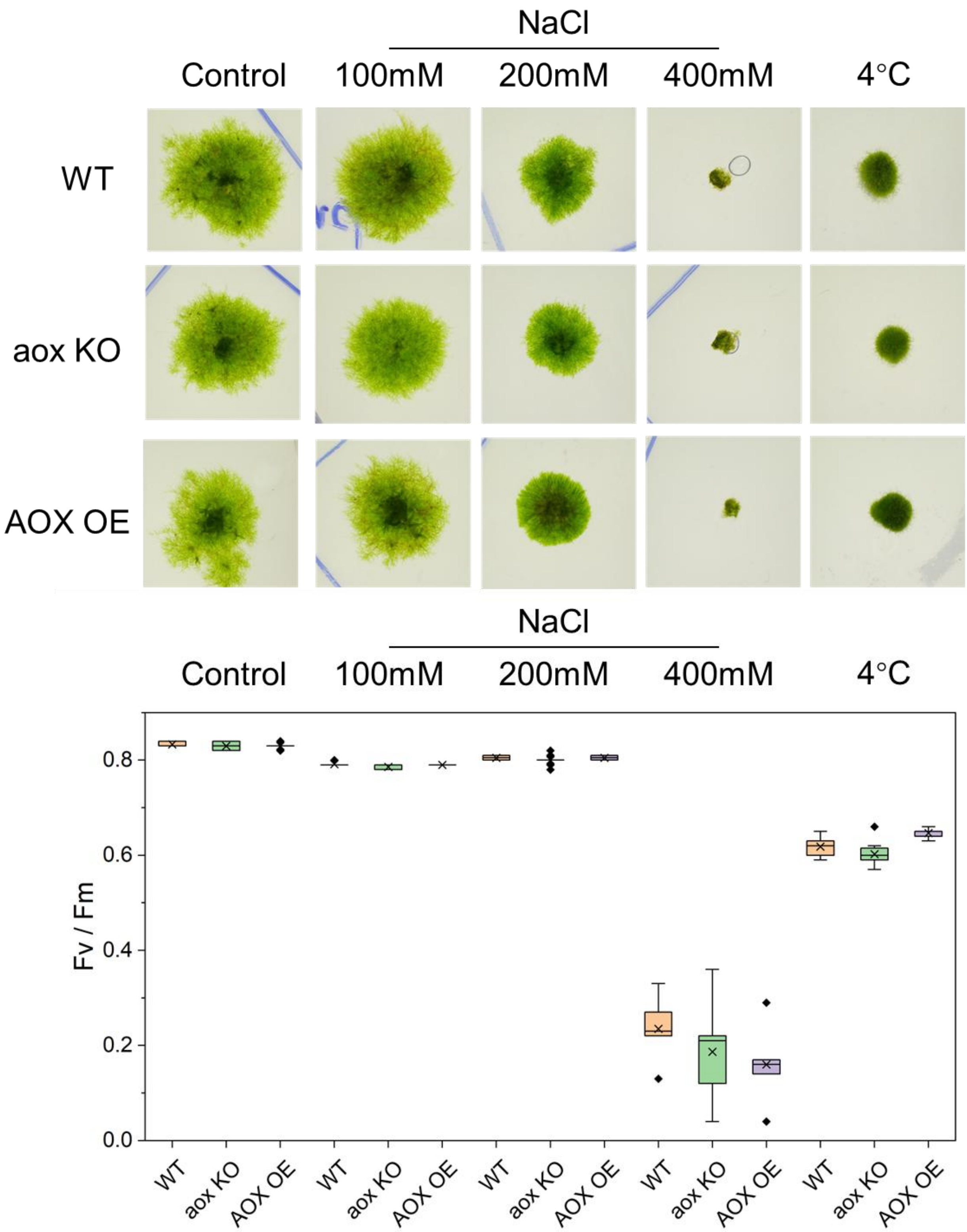

Figure S3 Salt and cold stress growth phenotypes of *P. patens* WT, *aox* KO lines and AOX OE lines. 10-day-old protonema colonies with a diameter of about 2 mm were transferred and grown under control light (50 μmol photons m^{-2} s^{-1}) on $PPNO_3$ medium supplemented with 100, 200, 400 mM NaCl or placed at 4°C. Photographs were taken and PSII maximum efficiency (*Fv/Fm*) was measured after 21-day growth. Bar = 2 mm. Data are shown as average ± SD (n=6). The asterisk indicates statistically significant differences (Independent sample test, $P<0.05$).

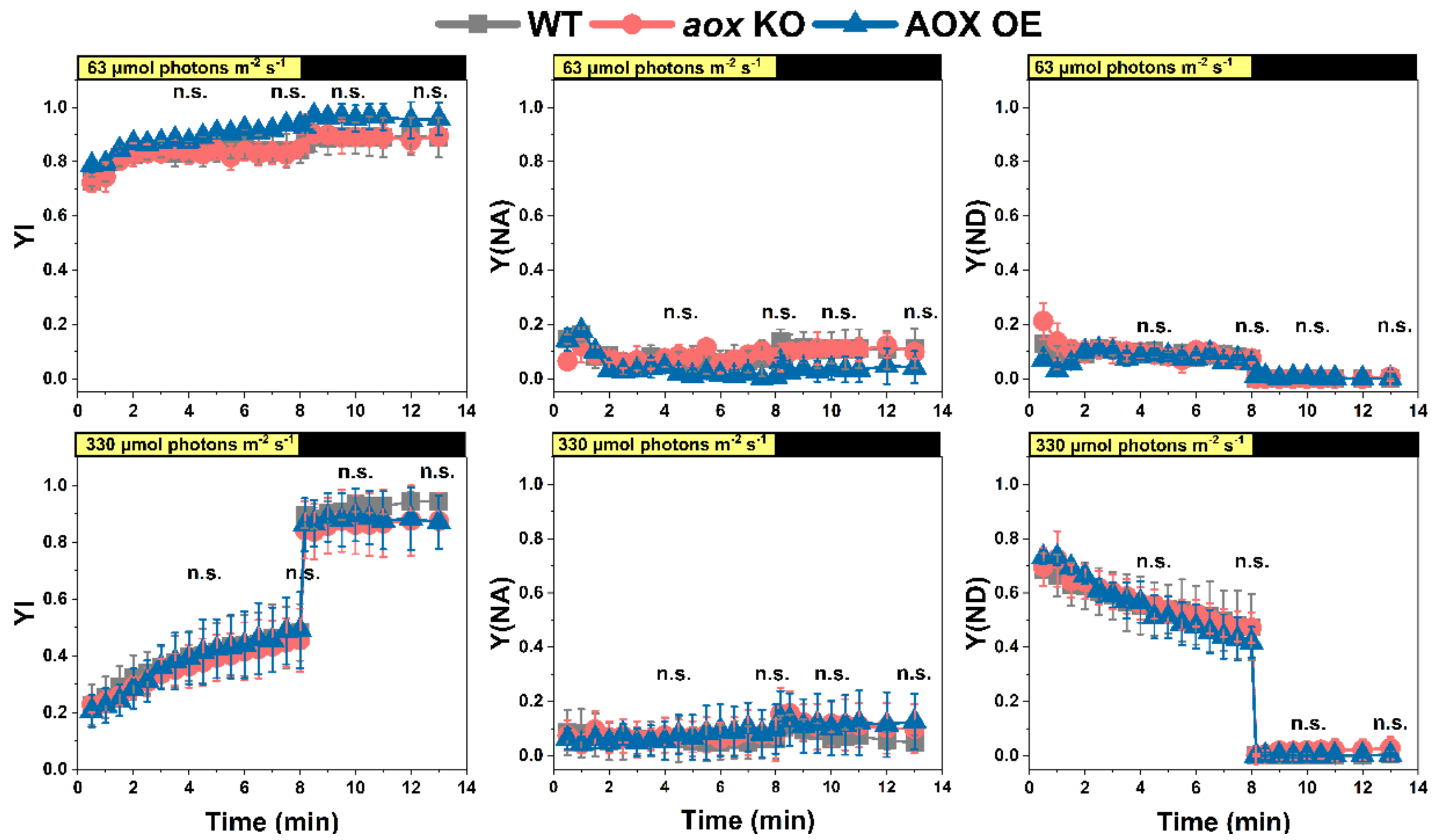

Figure S4 PSI photosynthetic characteristics of WT, *aox* KO, AOX OE protonema at different light intensities. 10-day-old protonema grown under control light (50 µmol photons m^{-2} s^{-1}) were measured with Dual PAM 100. After 40 min dark adaptation, plants were exposed to 63 µmol photons m^{-2} s^{-1} or 330 µmol photons m^{-2} s^{-1} actinic light for 8 min (indicated in yellow, top bar) followed by 5 min darkness (indicated in black, top bar). WT, *aox* KO and AOX OE are shown in black square, red cycle, and blue triangle, respectively. Data are shown as average ± SD (n=4). The n.s. indicate statistically no significant differences of three plants (one-way ANOVA, P>0.05) after 4, and 8 min of illumination and after 2 and 5 min in the dark. *aox* KO and AOX OE reports pooled data of at least two independent lines.

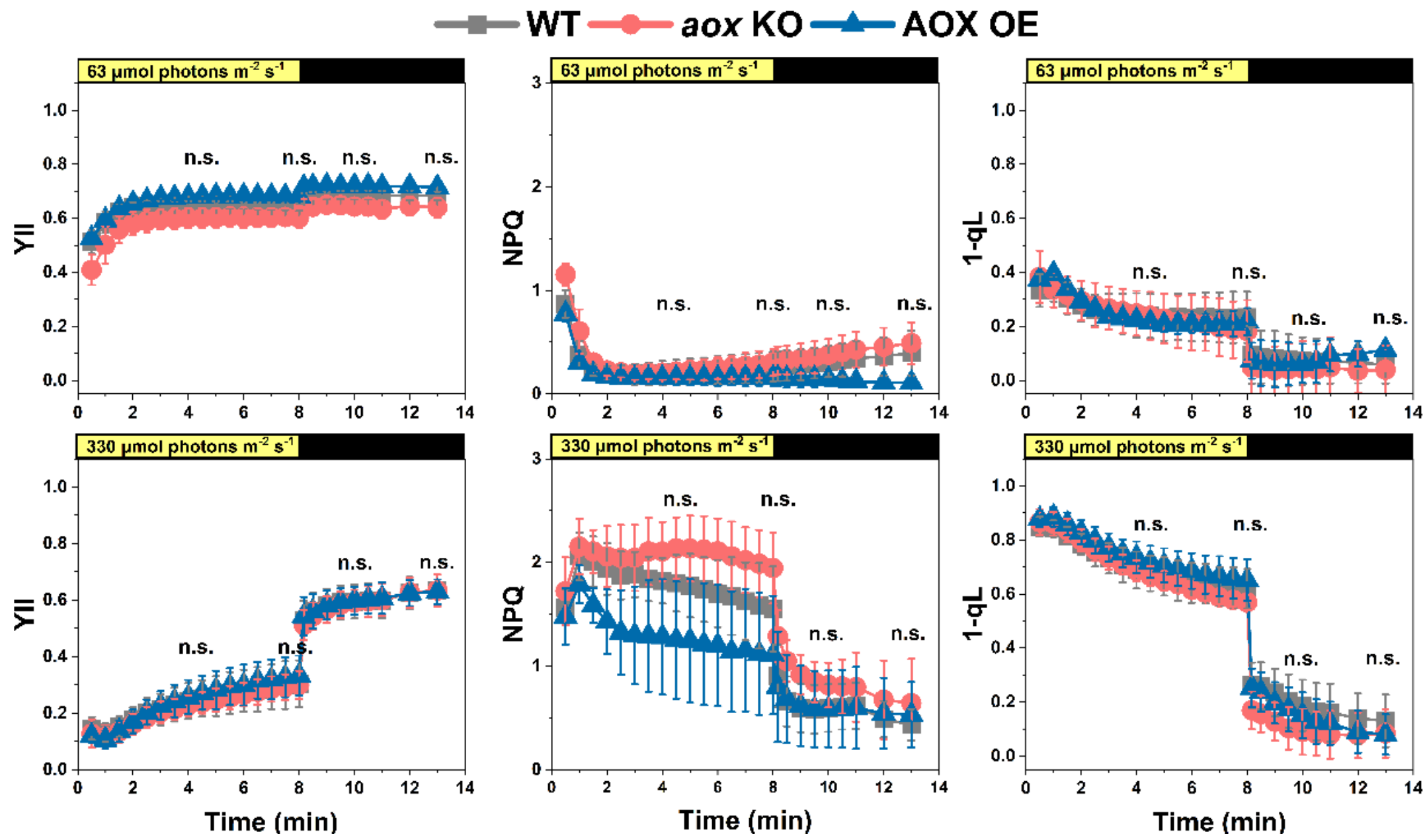

Figure S5 PSII photosynthetic characteristics of WT, *aox* KO, AOX OE protonema at different light intensities. 10-day-old protonema grown under control light (50 μmol photons m^{-2} s^{-1}) were measured with Dual PAM 100. After 40 min dark adaptation, plants were exposed to 63 μmol photons m^{-2} s^{-1} or 330 μmol photons m^{-2} s^{-1} actinic light for 8 min (indicated in yellow, top bar) followed by 5 min darkness (indicated in black, top bar). WT, *aox* KO, AOX OE are shown in black square, red cycle, and blue triangle, respectively. Data are shown as average ± SD (n=4). The n.s. indicate statistically no significant differences of three plants (one-way ANOVA, P>0.05) after 4, and 8 min of illumination and after 2 and 5 min in the dark. *aox* KO and AOX OE reports pooled data of at least two independent lines.

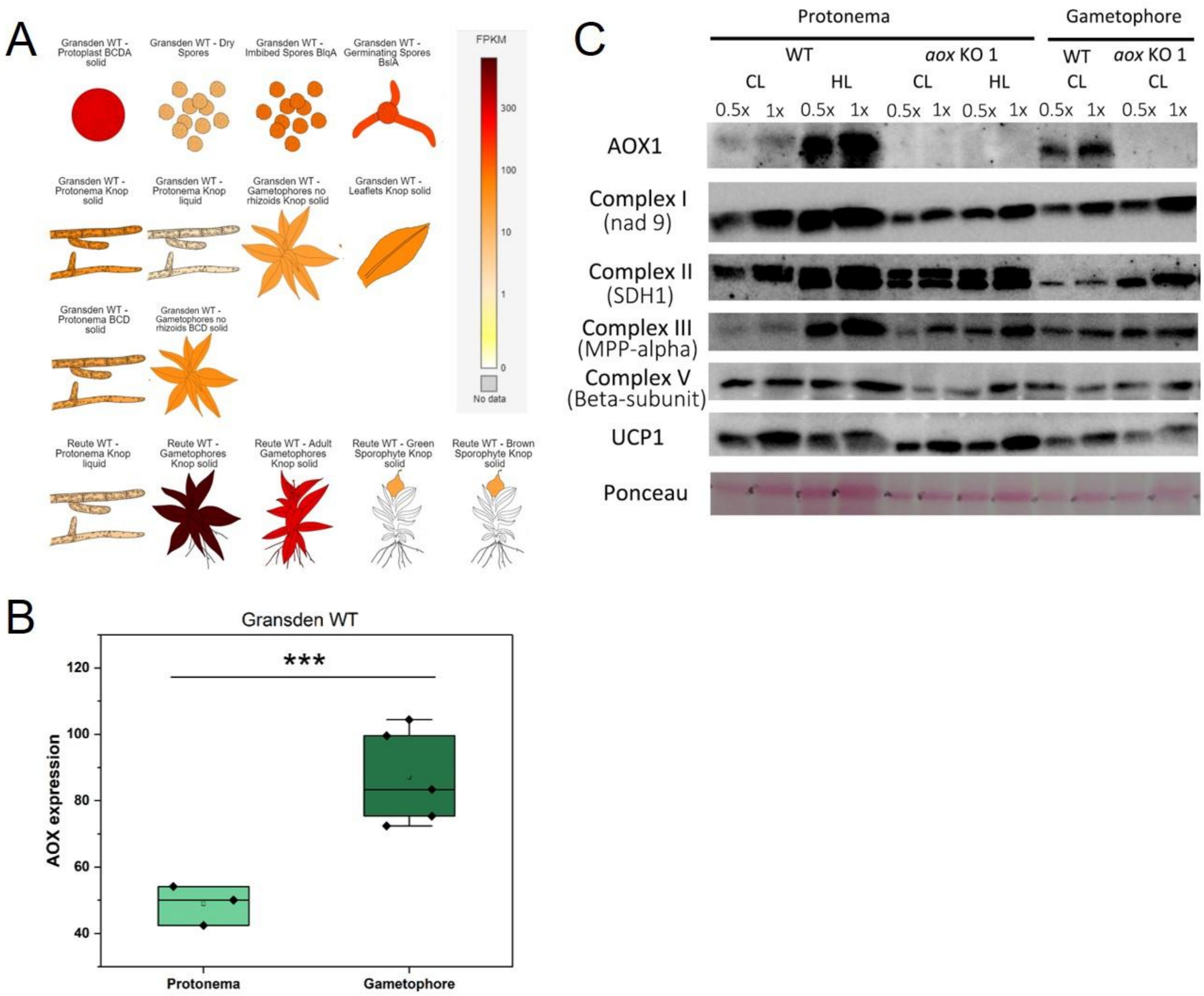

Figure S6 AOX expression in different developmental stages of *P.patens*. A. AOX gene expression map describing the transcript expression patterns of AOX during development in *P.patens* Gransden and Reute ecotypes. The graph was recomposed from PEAT moss https://peatmoss.plantcode.cup.uni-freiburg.de/. B. Transcript expression of the AOX in protonema and gametophore in *P.patens* Gransden. Data was downloaded from PEAT moss. The asterisk indicates statistically significant differences (Independent sample test, $P<0.01$) C. The amounts of protein on respiratory electron transport chain in protonema and gametophore. 10-day-old protonema and 2-month-old gametophore were grown on $PPNO_3$ medium under control light. Total protein extraction were loaded, 1x loading corresponded to 2 ug of total chlorophyll.

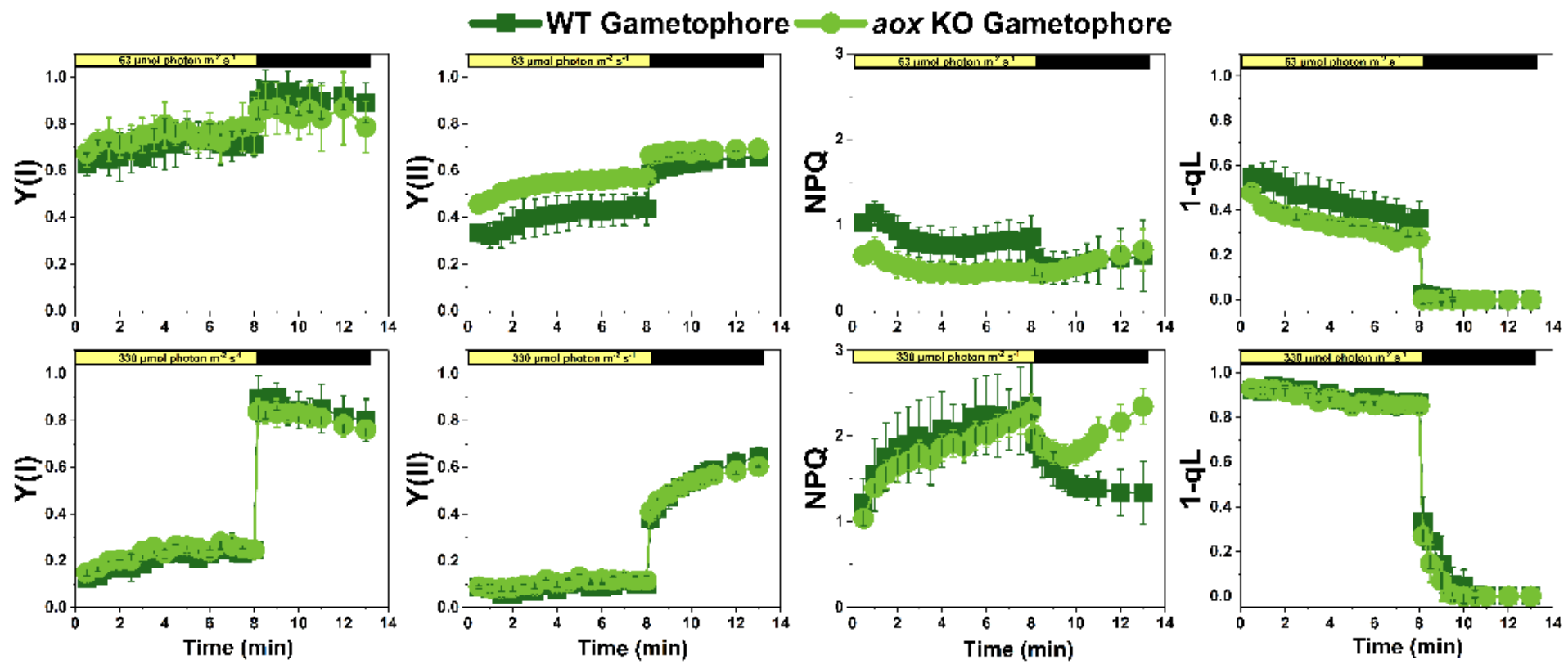

Figure S7 Photosynthetic fluorescence parameters in *P.patens* gametophores. Gametophores were cultivated on $PPNO_3$ medium at control light (50 µmol photons m^{-2} s^{-1}). 30-day-old gametophores were cut and measured at 63 µmol photons m^{-2} s^{-1} or 330 µmol photons m^{-2} s^{-1}. WT and *aox* KO lines are shown in dark green squares and light green cycles, respectively. Average ± SD (n=4) are reported. *aox* KO and AOX OE reports pooled data of at least two independent lines.

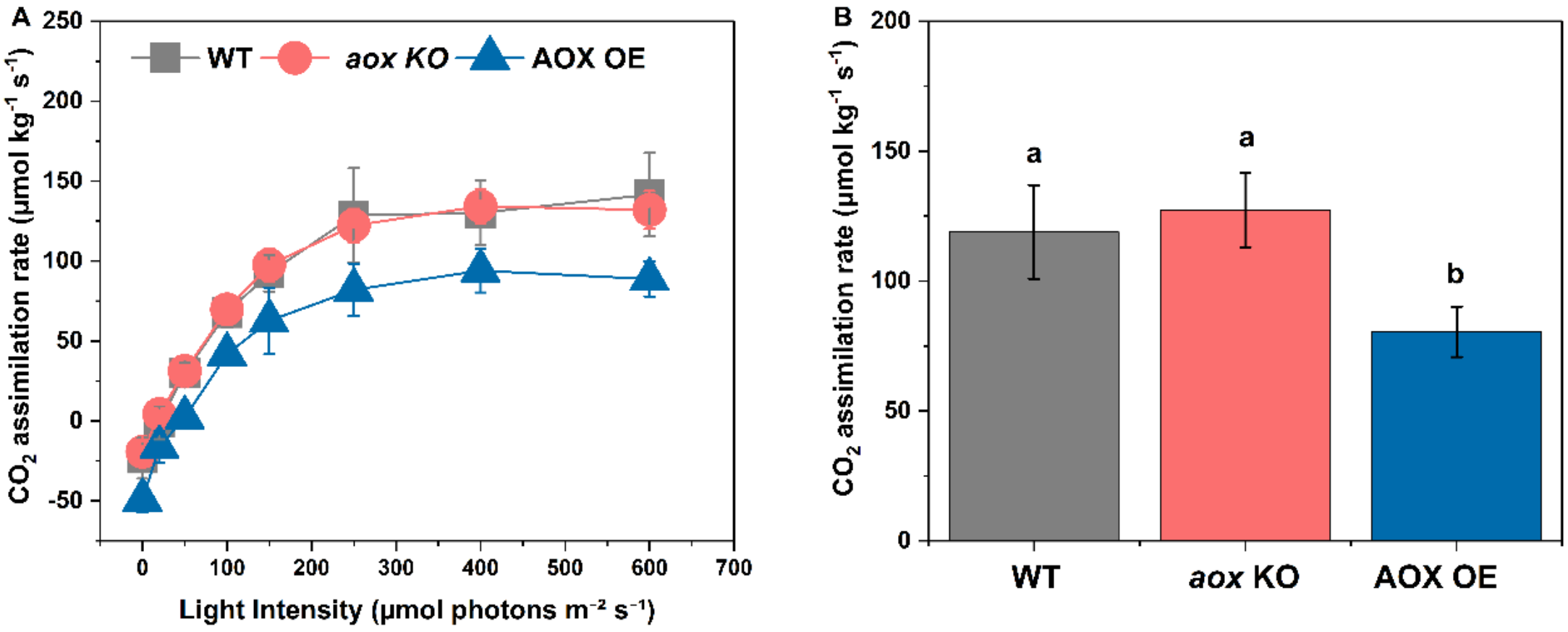

Figure S8 CO_2 fixation capacity of *Physcomitrium patens* grown under high light. A, Light response curve of CO_2 assimilation rate. B, Steady-state CO_2 assimilation rate at saturated light intensity (300 μmol photons m^{-2} s^{-1}). Gametophores were cultivated on $PPNO_3$ medium at high light (500 μmol photons m^{-2} s^{-1}). CO_2 assimilation rate was measured after 30 days of growth. WT, *aox* KO, and AOX OE lines are shown in black, red, and blue, respectively. Average ± SD (n=4) are reported. The different letters indicate statistically significant differences in WT, *aox* KO plants and AOX OE plants, respectively (one-way ANOVA, $P< 0.05$). *aox* KO and AOX OE reports pooled data of at least two independent lines.

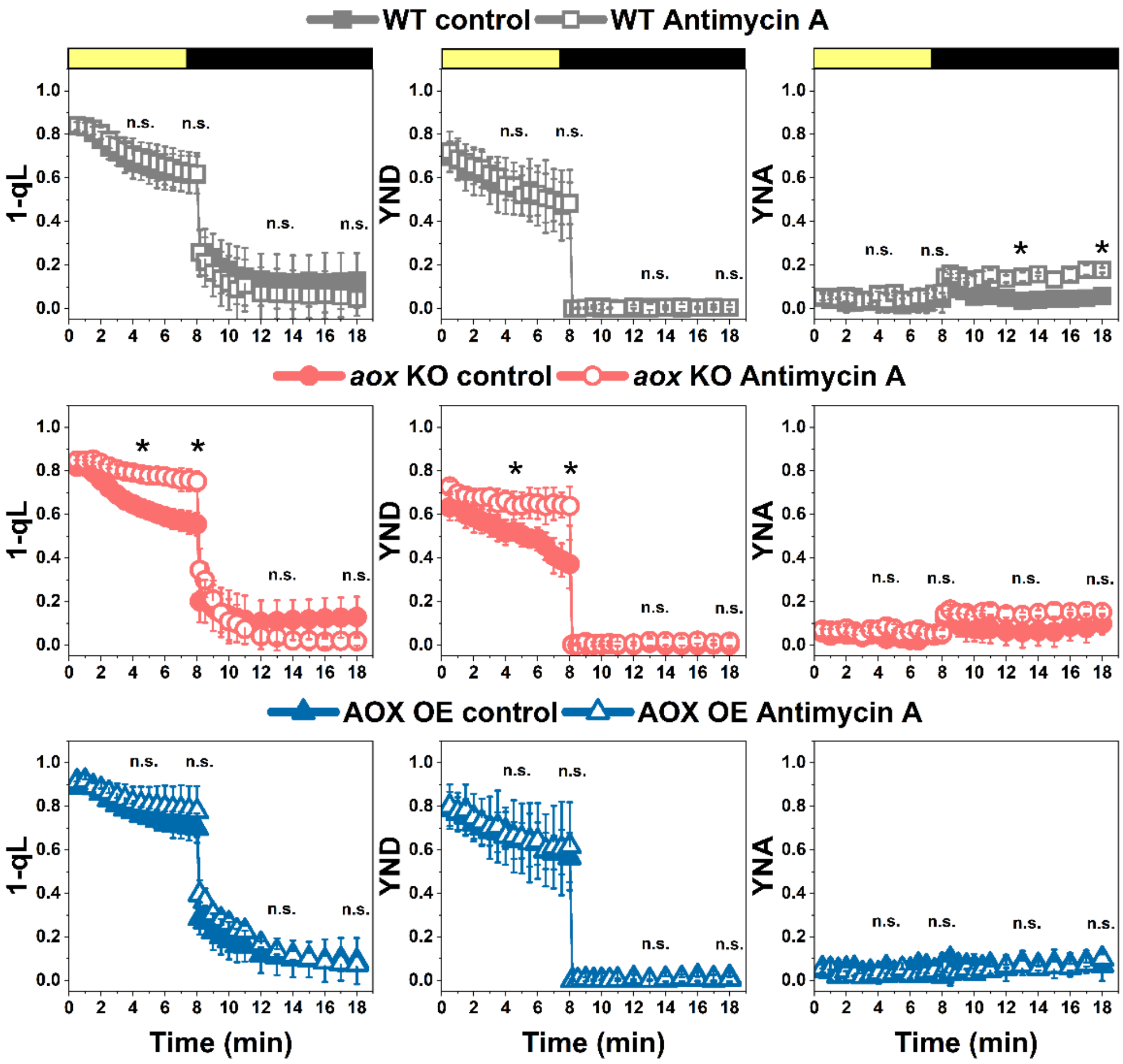

Figure S9 PQ and PSI redox state of WT, *aox* KO, AOX OE lines in *P.patens* protonema treated with antimycin A. 10-day-old protonema grown at control light (50 μmol photons m^{-2} s^{-1}) and were measured with Dual PAM 100. Plants were submerged with HEPES buffer (control) or 16 μM antimycin A during the 40 min dark adaptation, then plants were exposed to 330 μmol photons m^{-2} s^{-1} actinic light for 8 min (indicated in yellow, top bar) followed by 10 min darkness (indicated in black, top bar). WT, *aox* KO, and AOX OE plants are shown in black square, red cycle, and blue triangle respectively. Data are shown as average ± SD (n=4). The different symbols indicate statistically significant differences of the three plants (one-way ANOVA, asterisk: $P<0.05$, n.s.: $P>0.05$) after 4, and 8 min of illumination and after 5 and 10 min in the dark. *aox* KO and AOX OE reports pooled data of at least two independent lines.

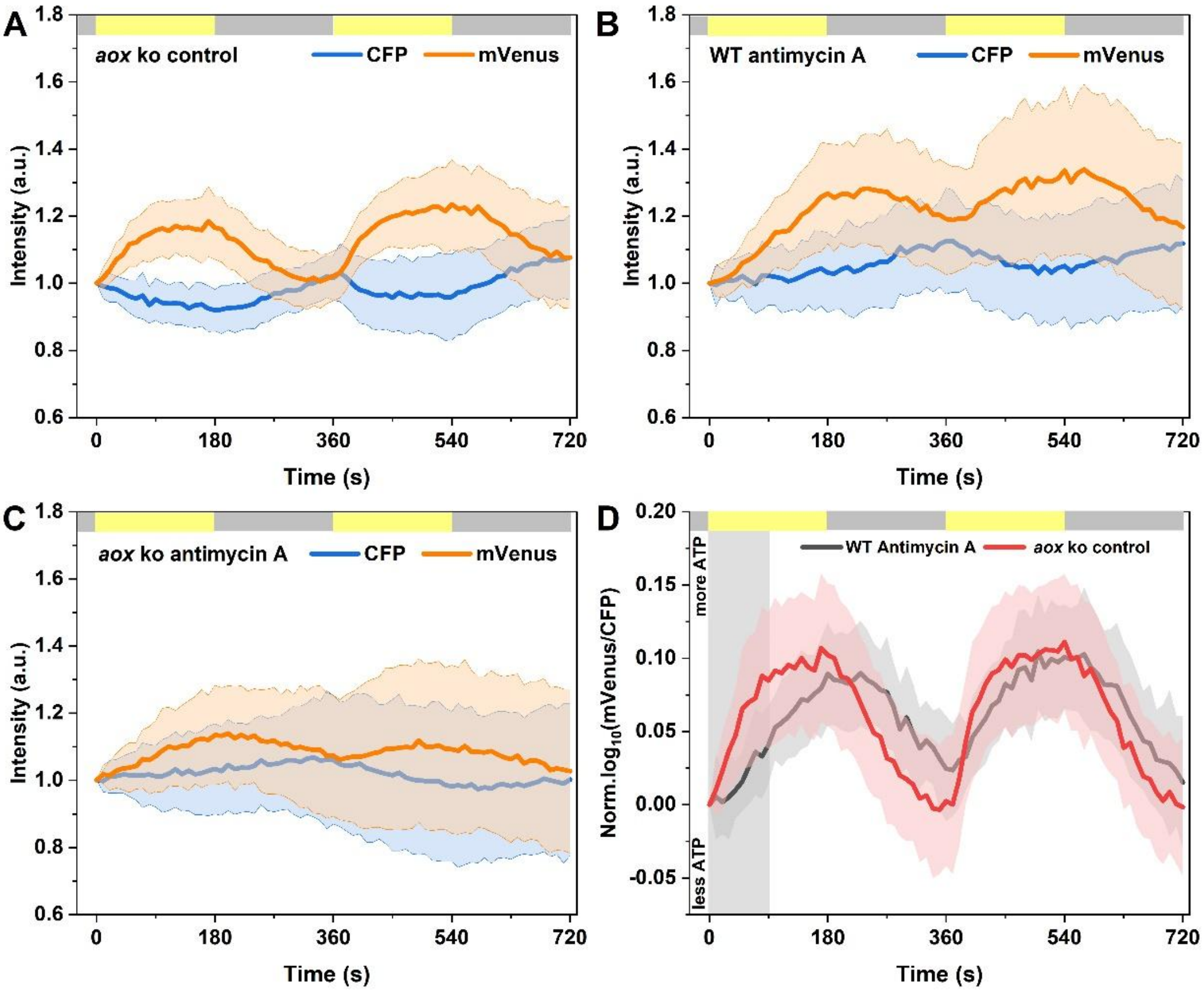

Figure S10 The contribution of complex III and AOX to light-induced $MgATP^{2-}$ accumulation in the cytosol of *Physcomitrium patens*. Normalized intensities of the single fluorescence channels of *aox*-cATeam plants control (A), WT-cATeam (B) *aox*-cATeam plants (C) treated with 16 µM antimycin A, showing their changes upon dark-to-light transitions. Plants were dark-adapted for 1 hour before the measurements, and the first data point was measured before switching light on. Light periods are marked with a yellow bar and correspond to 50 µmol photons m^{-2} s^{-1} of red light. Fluorescence channels are shown in Orange (mVenus) and cyan (CFP) together with experimental deviation (n = 58 ROIs from 3 independent samples in *aox*-cATeam control; n = 25 ROIs from 3 independent samples in antimyxin A treated WT-cATeam; *n* = 30 ROIs from 3 independent samples in antimycin A treated *aox*-cATeam). D) Normalized log-transformed FRET ratio during dark-to-light transitions of WT-cATeam plants treated with antimycin A (red) and *aox*-cATeam plants (black).

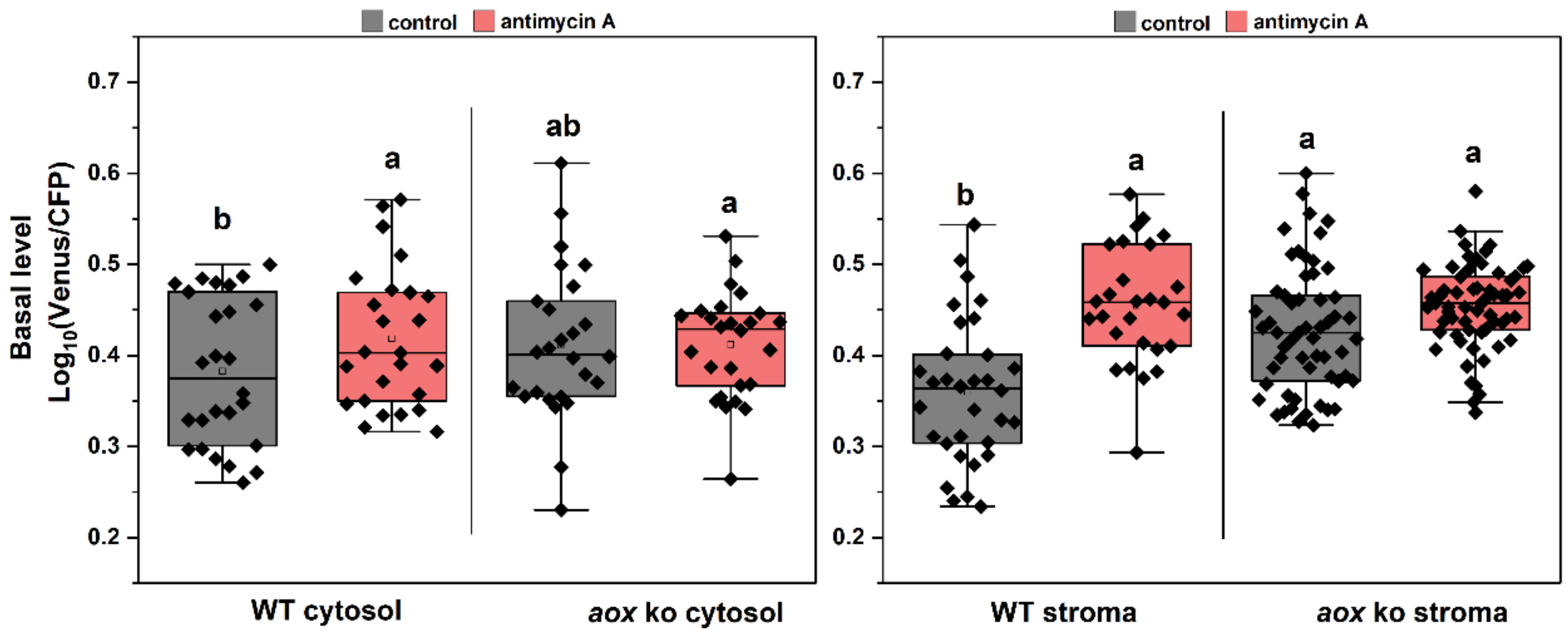

Figure S11 Comparison of unnormalized chloroplast stroma and cytosolic FRET ratio at basal level. The data was the average of three points measured before switching light on. The different letters indicate statistically significant differences in WT plants and *aox* ko plants, respectively (one-way ANOVA, $P < 0.05$).

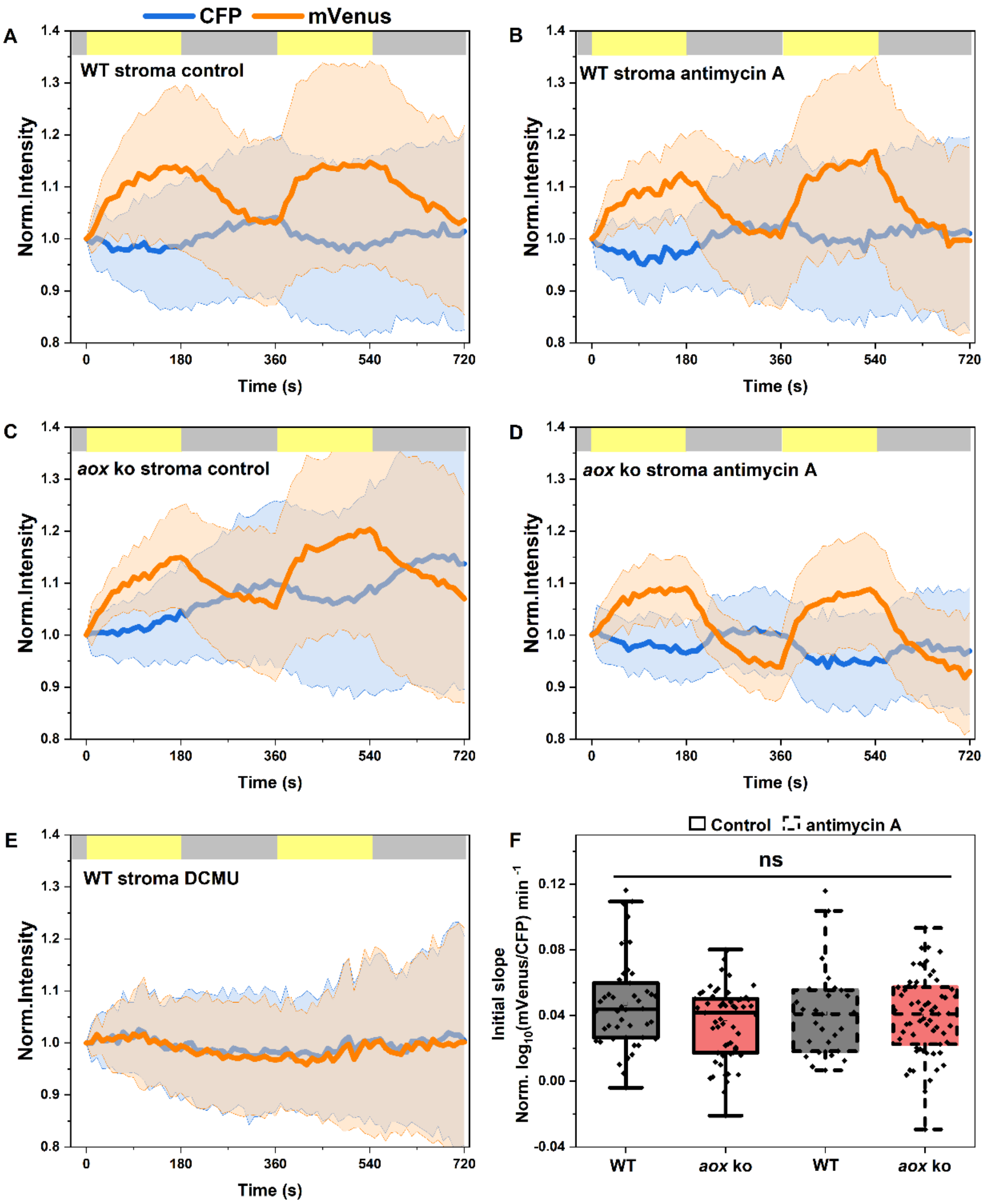

Figure S12 Single channels dynamics in the plastid of *Physcomitrium patens*. A-E, Normalized intensities of the single channels of WT-pATeam and *aox*-pATeam lines showing their changes upon dark-to-light transitions. Plants were dark-adapted for 1 hour before the measurements, and the first data point was measured before switching light on. Light periods are marked with a yellow bar and correspond to 50 μmol photons m^{-2} s^{-1} of red light. F, Slope during the first 90s of illumination, marked as shadow in the figure 9. The n.s. indicates statistically no differences between WT-pATeam and *aox*-pATeam lines under control and treatment, respectively (one-way ANOVA, $P < 0.05$).

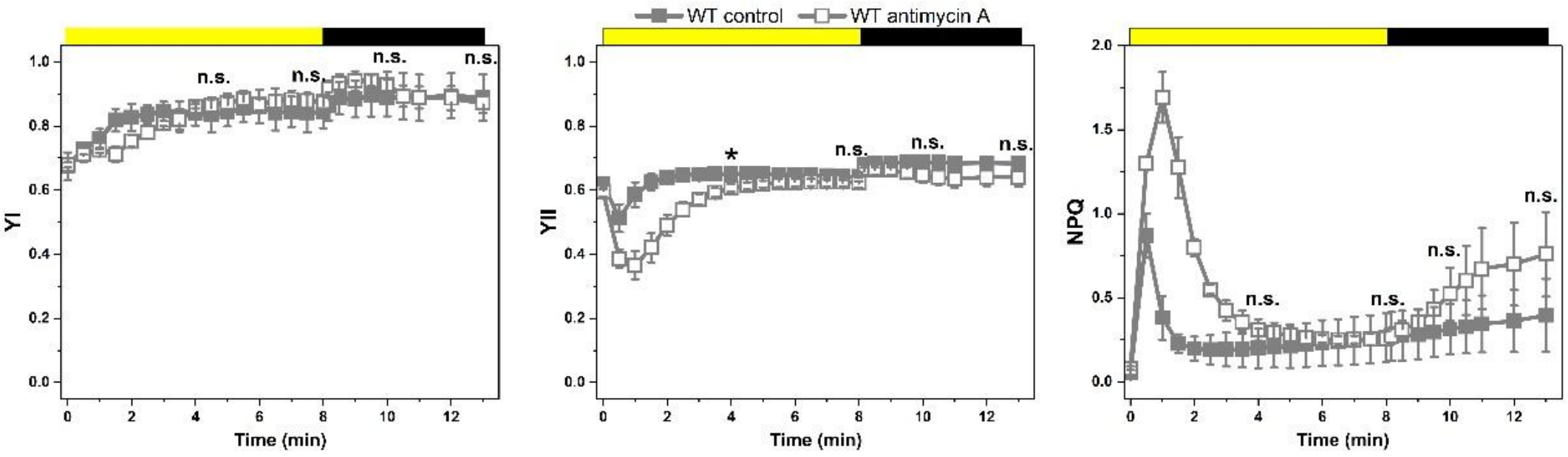

Figure S13 Photosynthetic propoters at growth light in *P.patens* protonema treated with antimycin A. 10-day-old WT protonema grown at control light (50 µmol photons m^{-2} s^{-1}) and were measured with Dual PAM 100. Plants were submerged with HEPES buffer (control) or 16 µM antimycin A during the 40 min dark adaptation, then plants were exposed to 50 µmol photons m^{-2} s^{-1} actinic light for 8 min (indicated in yellow, top bar) followed by 5 min darkness (indicated in black, top bar). Control and antimycin A treated samples were shown as solid square and empty square respectively. Data are shown as average ± SD (n = 4 or 3). The different symbols indicate statistically significant differences of the three plants (Independent sample test, asterisk: P<0.05, n.s.: P>0.05) after 4, and 8 min of illumination and after 2 and 5 min in the dark. *aox* KO and AOX OE reports pooled data of at least two independent lines.
